# Trophic transfer of polyunsaturated fatty acids across the aquatic–terrestrial interface: An experimental tritrophic food chain approach

**DOI:** 10.1002/ece3.9927

**Published:** 2023-03-24

**Authors:** Katharina Ohler, Verena C. Schreiner, Dominik Martin‐Creuzburg, Ralf B. Schäfer

**Affiliations:** ^1^ iES Landau Institute for Environmental Sciences, RPTU Kaiserslautern‐Landau Fortstraße 7 76829 Landau in der Pfalz Germany; ^2^ Department of Aquatic Ecology BTU Cottbus‐Senftenberg Research Station Bad Saarow Cottbus Germany

**Keywords:** aquatic‐terrestrial, PUFA, trophic transfer

## Abstract

Aquatic and their adjacent terrestrial ecosystems are linked via the flux of organic and inorganic matter. Emergent aquatic insects are recognized as high‐quality food for terrestrial predators, because they provide more physiologically relevant long‐chain polyunsaturated fatty acids (PUFA) than terrestrial insects. The effects of dietary PUFA on terrestrial predators have been explored mainly in feeding trials conducted under controlled laboratory conditions, hampering the assessment of the ecological relevance of dietary PUFA deficiencies under field conditions. We assessed the PUFA transfer across the aquatic–terrestrial interface and the consequences for terrestrial riparian predators in two outdoor microcosm experiments. We established simplified tritrophic food chains, consisting of one of four basic food sources, an intermediary collector gatherer (*Chironomus riparius*, Chironomidae), and a riparian web‐building spider (*Tetragnatha* sp.). The four basic food sources (algae, conditioned leaves, oatmeal, and fish food) differed in PUFA profiles and were used to track the trophic transfer of single PUFA along the food chain and to assess their potential effects on spiders, that is, on fresh weight, body condition (size‐controlled measurement of nutritional status), and immune response. The PUFA profiles of the basic food sources, *C. riparius* and spiders differed between treatments, except for spiders in the second experiment. The PUFA α‐linolenic acid (ALA, 18:3n‐3) and ɣ‐linolenic acid (GLA, 18:3n‐6) were major contributors to the differences between treatments. PUFA profiles of the basic food sources influenced the fresh weight and body condition of spiders in the first experiment, but not in the second experiment, and did not affect the immune response, growth rate, and dry weight in both experiments. Furthermore, our results indicate that the examined responses are dependent on temperature. Future studies including anthropogenic stressors would deepen our understanding of the transfer and role of PUFA in ecosystems.

## Graphical abstract

Aquatic and their adjacent terrestrial ecosystems are linked via the flux of organic and inorganic matter. We assessed the PUFA transfer across the aquatic‐terrestrial interface and the consequences for terrestrial riparian predators in outdoor microcosm experiments. We established simplified tritrophic food chains, consisting one basic food source, an intermediary collector gatherer (*Chironomus riparius*, Chironomidae), and a riparian web‐building spider (*Tetragnatha* sp.). The four basic food sources (algae, conditioned leaves, oatmeal, fish food) differed in PUFA profiles and were used to track the trophic transfer of single PUFA along the food chain and to assess their potential effects on spiders.

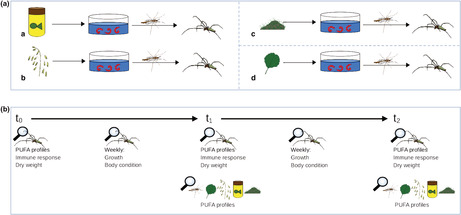

## 1 INTRODUCTION

Natural ecosystems are in exchange with neighboring ecosystems. Aquatic and adjacent terrestrial ecosystems are linked via the exchange of organic and inorganic matter (Baxter et al., 2005; Schindler & Smits, 2017). Leaves and terrestrial invertebrates falling into streams can be an important subsidy for stream food webs (Baxter et al., 2005; Wallace et al., 1997). In turn, emergent aquatic insects can be a food source for terrestrial predators, such as bats, lizards, and spiders (Kato et al., 2004; Sabo & Power, 2002; Sullivan et al., 1993). In particular, when terrestrial food sources are scarce, for example, in spring, terrestrial predators can benefit from feeding on emergent aquatic insects (Nakano & Murakami, 2001; Wesner, 2010). Besides food quantity, food quality is key for understanding energy fluxes between ecosystems and for predicting effects on subsidized food webs (Marcarelli et al., 2011).

Emergent aquatic insects are considered high‐quality food for terrestrial predators, because they contain up to 10 times more long‐chain (≥20 carbon atoms) polyunsaturated fatty acids (PUFA) than terrestrial insects (Hixson et al., 2015; Parmar et al., 2022). Aquatic primary producers, such as diatoms or cryptophytes, can synthesize long‐chain PUFA that are subsequently available to aquatic consumers and transferred across trophic levels (Ahlgren et al., 1992; Kainz et al., 2004; Strandberg et al., 2015). In contrast, terrestrial primary producers typically do not produce long‐chain PUFA (Sayanova & Napier, 2004). Most consumers are incapable of synthesizing long‐chain PUFA de novo and thus rely on an adequate dietary supply with these essential compounds (Twining, Bernhardt, et al., 2021). Long‐chain PUFA, such as eicosapentaenoic acid (EPA, 20:5n‐3), docosahexaenoic acid (DHA, 22:6n‐3), and arachidonic acid (ARA, 20:4n‐6), are important membrane components and serve as precursors for a plethora of other bioactive molecules. However, some animals, for example, some spiders and birds, are able to synthesize C20 PUFA from dietary C18 PUFA precursors, that is, α‐linolenic acid (ALA, 18:3n‐3) and linoleic acid (LIN, 18:2n‐6c), which is energetically costly and thus may be performed only if required (Mathieu‐Resuge et al., 2022; Twining, Bernhardt, et al., 2021; Twining, Parmar, et al., 2021). Riparian predators may thus benefit from the consumption of emergent aquatic insects (Fritz et al., 2017; Mayntz & Toft, 2001; Twining et al., 2016). PUFA‐rich food was shown to promote growth, immune responses, and good body condition of tree swallow chicks (Twining et al., 2016), as well as growth (Mayntz & Toft, 2001) and immune responses of spiders (Fritz et al., 2017).

The transfer of PUFA from aquatic into adjacent terrestrial ecosystems via emergent aquatic insects may thus strongly influence riparian food webs. Due to their important role in physiological processes, long‐chain PUFA are typically stored in tissues without greater modifications. Thus, PUFA tend to bioaccumulate and are nearly twice as efficiently transferred to the next trophic level as bulk carbon (Arts et al., 2001; Brett & Müller‐Navarra, 1997; Gladyshev et al., 2013).

Previous studies examined the PUFA profiles of aquatic insects (Martin‐Creuzburg et al., 2017; Moyo et al., 2017; Scharnweber et al., 2020), the PUFA transfer via aquatic insects to terrestrial predators (Kowarik et al., 2021; Twining et al., 2019; Twining, Parmar, et al., 2021), and effects of dietary PUFA on terrestrial predators in the field (Fritz et al., 2017) as well as under controlled laboratory conditions (Mayntz & Toft, 2001; Twining et al., 2016, 2019). For the latter, it remains open to which extent the results can be transferred to the field because environmental factors such as temperature can also affect the performance of predators, for example, regarding growth (Brown et al., 2004) and immune function (Wojda, 2017).

Our aim was to examine how PUFA are transferred along experimental tritrophic food chains and to explore potential effects on a terrestrial predator. The latter was studied in outdoor microcosms under virtually realistic environmental conditions, that is, the spiders were exposed to normal weather conditions and had the possibility to build orb webs on nettles like in their natural habitat. Overall, we conducted two outdoor microcosm experiments each with basic food sources, the *Chironomus riparius* (Diptera, Chironomidae) and the spider *Tetragnatha* sp. in a food chain. *Tetragnatha* sp. are suitable model organisms to study direct and indirect effects of PUFA in aquatic–terrestrial food webs because they commonly occur in riparian areas, consume aquatic emergent insects (Kato et al., 2004), and capture their prey with orb webs (Reitze & Nentwig, 1991). Furthermore, it was shown that spiders aggregate in riparian areas during peak emergence of aquatic insects (Henschel et al., 2001; Paetzold et al., 2011) and potentially serve as prey for other organisms such as birds (Poulin et al., 2010), which may result in a PUFA transfer via spiders to higher trophic levels.

The food chains varied in the basic food source, which were selected to represent different PUFA profiles enabling us to determine potential differences in their transfer and effects on spiders. Therefore, PUFA profiles of the basic food sources, *C. riparius*, and spiders were analyzed. Additionally, we recorded fresh and dry weight, growth rate, body condition (size‐controlled measurement of nutritional status), and immune response of spiders to assess potential effects of PUFA on spider performance. We expected (1) different PUFA profiles between treatments at all trophic levels of the food chain, because differences in PUFA profiles of the basic food sources can propagate along the food chain; (2) temporal changes in PUFA profiles of the spiders during the experiment, due to the fact that PUFAs are assimilated after a certain time into the tissue of organisms; and (3) treatment differences in the spiders' fresh and dry weight, growth rate, body condition, and immune response due to the differences in PUFA profiles of the chironomids consumed by the spiders.

## 2 MATERIALS AND METHODS

### 2.1 Spider and nettle collection

For the outdoor microcosm experiments, spiders of the genus *Tetragnatha* were collected at pristine streams (49°16′13″N, 8°2′54″E and 49°15′43″N, 7°57′36″E) in the Palatinate Forest Nature Park, a forested low mountain range in Germany. The collection for the first experiment was conducted on the 17th, 23rd, 24th, and 25th of April 2019 and for the second experiment on the 17th, 19th, and 24th of June 2019. Until the start of the experiment, the spiders were kept in climate chambers at 20°C and were fed once a week with one adult *C. riparius* raised in standard cultures (OECD, 2011).

Feeding frequency varies between and within spider species (Foelix, 2011) and the immune response of male and pregnant female spiders can be highly variable (Ahtiainen et al., 2005, 2004). Therefore, whenever possible, only female, adult and nonpregnant (checked visually) spiders of the species *T. montana* were used in the experiment to minimize variability in their feeding and immune response (Table S1).

Nettles, *Urtica dioica*, were used in the microcosms because they are common along streams (Davis, 1989) and are frequently used by spiders in their natural habitat to build their orb webs. Nettles were collected at two locations (49°16′57″N, 8°5′18″E and 49°12′15″N, 8°6′27″E) on the 10th, 11th, 16th, 24th, and 29th of April 2019. After collection, nettles were planted in fertilized soil (nitrogen 150–450 mg L^−1^) in 10 cm × 10 cm × 10 cm pots and kept in 60 cm × 60 cm × 90 cm aerarium (Matthäus Hahn e.K.) until the experiment started to prevent insect infestation. Furthermore, the microcosms were regularly checked for invading insects, which were removed by hand.

### 2.2 Chironomidae and basic food sources

Adult *C. riparius*, hereafter chironomids, were used as food for the spiders. Chironomids were gathered from laboratory cultures that were maintained based on the OECD‐guideline 235 (OECD, 2011); they were cultured in glass vessels (30 cm × 20 cm × 10 cm) with a layer of silica sand (height ~ 0.1 cm) in a climate chamber (20 ± 2°C) with a 16‐h light (~1000 lux) and 8‐h darkness light cycle.

Chironomid cultures differing in the food source for chironomid larvae, termed as basic food sources below, were set up at the end of February 2019 to gain organisms with specific PUFA profiles. The early setup of the cultures was made, to ensure that at the beginning of the experiment, the chironomids had only consumed the specific basic food sources during their larval stages. Overall, four basic food sources were used and later the treatments of the experiments are named after the basic food sources.

The basic food sources were provided ad libitum and were algae (Liquizell, Hobby), standard fish food (TetraMin, Tetra), oatmeal (*Avena sativa*, dmBio), and conditioned alder leaves (*Alnus glutinosa*). The latter three were ground before being fed to the chironomids. Fish food is used in the OECD‐guideline 235 (OECD, 2011) to feed chironomids as it comprises a suitable nutrient composition for chironomids. Furthermore, fish food includes ingredients with aquatic and terrestrial origin among others fish and cereals and hence should have a broad PUFA profile. Algae was used as aquatic food source and contained phytoplankton and minerals, which usually is enriched in n‐3 PUFA in comparison with n‐6 PUFA (Strandberg et al., 2015). In contrast, oatmeal represented a typical terrestrial food source, because it contains high levels of LIN and only small‐to‐no amounts of ALA and EPA (Torres‐Ruiz et al., 2010). Conditioned leaves were considered as semiaquatic food, because they originate from terrestrial plants, but are modified by aquatic organisms. Conditioned leaves can be an important food source for aquatic invertebrates, especially in small order streams (Graça & Canhoto, 2006; Vannote et al., 1980). Conditioned leaves are colonized by microorganisms such as aquatic fungi and bacteria as well as eukaryotic microalgae. Particularly, aquatic fungi can enhance the protein and lipid content of leaves and decompose otherwise hardly degradable leaf compounds, such as cellulose, rendering leaves more nutritious and digestible (Bärlocher, 1985). Additionally, aquatic fungi change the PUFA content of leaves (Zubrod et al., 2017). The leaves were collected directly from the tree in autumn 2017 from a biosphere reserve (49°14′24″N, 7°53′24″E) at the time of abscission, air‐dried, and stored in the dark at room temperature until usage. Approximately 8 g of leaves was weighed with a precision of 0.01 g in leaf bags (0.5 mm mesh size, 15 × 15 cm). Starting at the end of January 2019, every 2 weeks two leaf bags were conditioned in the Sulzbach in Eußerthal, Germany (49°15′43″N, 7°57′36″E).

### 2.3 Experimental design

We conducted two experiments on Campus Landau (49°12′15″N, 8°6′27″E) in southwest Germany. The first experiment took place from the 29th of April to the 12th of June 2019, and the second experiment was run from the 8th to 29th of July 2019. The duration of the two experiments differed, because during the second experiment, the mortality of spiders was elevated, probably due to very hot temperatures inside the microcosms of up to 32 ± 8°C (Figure S1).

**FIGURE 1:**
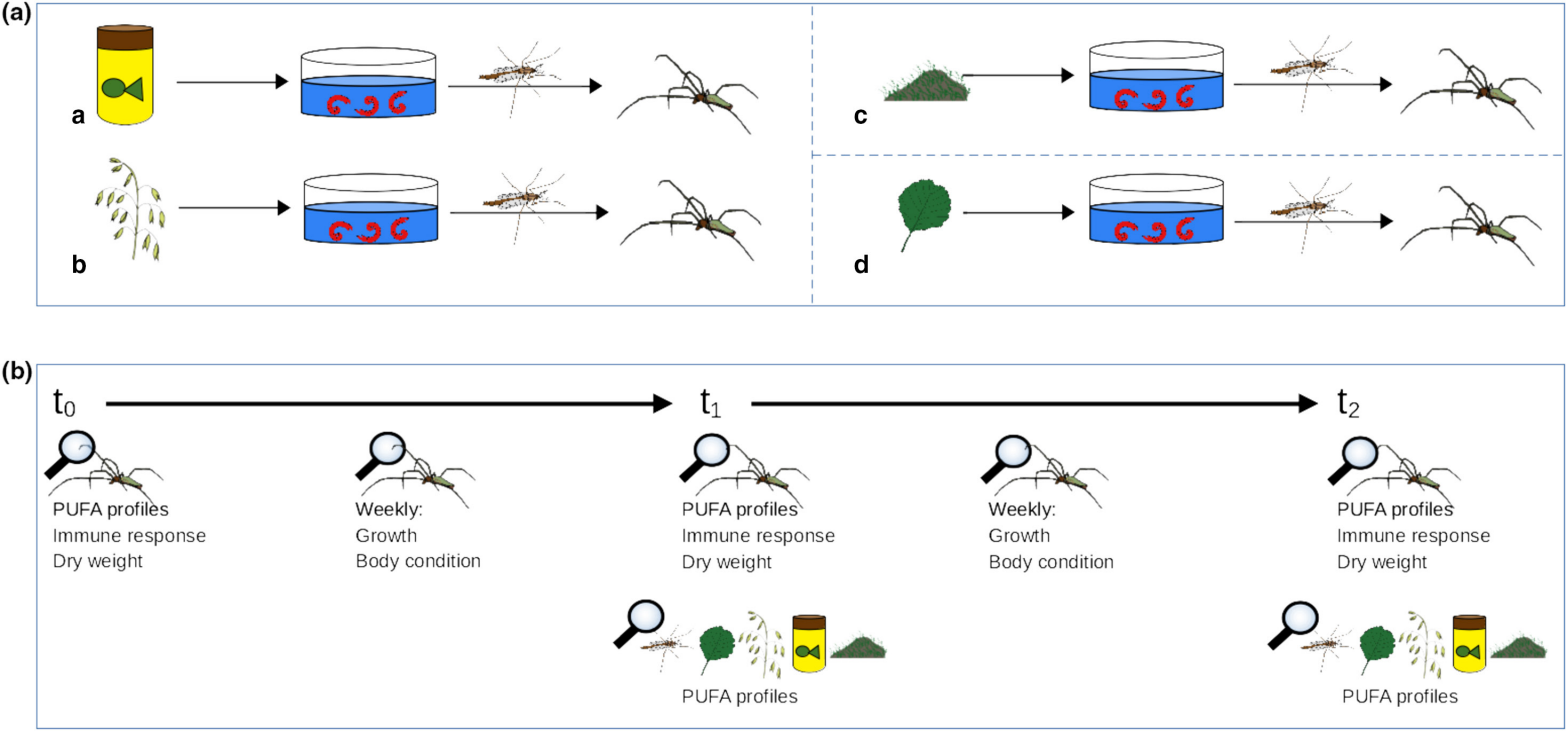
Experimental design. (a) Treatments of the experiments. Every treatment consisted of one food chain with a basic food source at the lowest trophic level, followed by chironomids at the second level and spiders on the highest trophic level. Basically, the treatments differed in the basic food source. A: Fish food; B: Oatmeal; C: Algae; D: Leaves. The first experiment included algae, fish food and oatmeal as basic food treatment, whereas the second experiment included leaves, fish food and oatmeal. (b) Time course of the microcosm experiments. *t*_0_: Start of experiment (day = 0); *t*_1_: 23 and 14 days in first and second experiment. *t*_2_: 44 and 21 days in first and second experiment. Between the time points *t*_0_, *t*_1_, *t*_2_, fresh weight and body condition of spiders were assessed weekly.

Every treatment consisted of one food chain with a basic food source at the lowest trophic level, followed by chironomids as the second level and spiders as the highest trophic level. The treatments differed only in the basic food source supplied to the chironomid larvae (Figure 1). The first experiment included algae, fish food, and oatmeal as basic food treatment, whereas the second experiment included leaves, fish food, and oatmeal. The basic food treatments differed between experiments due to limited labor capacity preventing us to run all treatments simultaneously. Per treatment, 20 replicates were used. Every replicate consisted of one spider and one nettle in a microcosm (60 × 60 × 90 cm aerarium). The spiders and nettles were assigned randomly to the microcosms.

Spiders were fed on 2 days per week with a tweezer to ensure the spiders consumed the chironomids. In the first experiment, spiders were fed two chironomids per week and in the second experiment four chironomids per week. The number of chironomids differed because the algae‐based chironomid cultures were less productive during the first experiment and therefore prohibited feeding the spiders with four chironomids, which presumably better matches the energy needs of spiders. For the first 2 weeks, dead spiders were replaced in both experiments. Before the replacement spiders were put in the microcosms, they were being fed with the total amount of chironomids the dead spider had consumed and over the same period the dead spider was fed to safeguard that the results are not influenced by different food quantities.

### 2.4 Weekly measurement of fresh weight, growth and body condition

Once per week, the spiders were weighed. This fresh weight was used to calculate the growth rate (g week^−1^) of the spiders, where *t*_x_ is the number of weeks of the experiment and *t*_0_ the start of the experiment:

(1) $$\begin{array}{c} \text{growth rate}=\frac{\ln\left({\text{fresh weight}}_{t_{x}}\right)-\ln\left({\text{fresh weight}}_{t_{0}}\right)}{t_{x}} \end{array}$$

Furthermore, a picture of every spider was taken on top of millimeter paper to estimate their body condition using their thorax and abdomen width. The thorax grows only by molting, while the abdomen is more dynamic than the thorax and changes its size during food uptake. As the spiders were fed the same amount during the experiment, the size change due to food uptake is supposed to be similar. The body condition is a better indicator of the nutritional status of a spider than body weight alone because the abdomen is the main food storage and its proportion of the total weight increases with increasing relative width to the thorax width (Anderson, 1974). Furthermore, the body condition controls for the size of spiders, which is important when organisms of different life stages like in our study are compared (Jakob et al., 1996). The program ImageJ version 1.53 k (Rasband, 2018) was used to measure the width. Subsequently, the body condition was calculated as follows (Anderson, 1974):

(2) $$\text{body condition}=\frac{\text{thorax width}}{\text{abdomen width}}$$

### 2.5 Measurement of immune response, dry mass, and PUFA profiles

In the beginning, during and at the end of the experiment, spiders were taken randomly out of the experiment to analyze the immune response, dry mass, and PUFA profiles of spiders. We planned to analyze 10 spiders per treatment and time point, which was hampered by a higher than expected mortality. Nevertheless, at least three spiders were taken per treatment and time point (for exact numbers see Table S2). Additionally, adult chironomid and basic food source samples (Table S2) were taken to facilitate the comparison of PUFA profiles along the food chain. We sampled chironomids approximately 1 week before the spiders to allow spiders to digest and assimilate PUFA from the consumed chironomids.

To estimate the immune response of the spiders, their ventral abdomen was punctuated with a sterile needle (gauge = 0.45 mm) and subsequently, a nylon monofilament (length ≈ 2 mm, diameter ≈ 0.2 mm) was inserted (Fritz et al., 2017; Rantala et al., 2002; Siva‐Jothy & Thompson, 2002). In spiders, the monofilaments cause the same encapsulation reaction as parasites (Ratcliffe & Rowley, 1979). By quantifying the hemocytes adhering to the monofilament, the level of encapsulation can be determined. 24 h after insertion, the spiders were euthanized in liquid nitrogen. The monofilament was recovered from the thawed spiders and stored in ethanol (70%). The spiders were stored at –80°C until further processing. Each monofilament was photographed under a binocular with millimeter paper in three random orientations. In the pictures, the encapsulated area (*A*_encapsulated_) and the area of the nonencapsulated monofilaments (*A*_not encapsulated_) were measured using ImageJ (Rasband, 2018). The ratio of these two areas was reported as proportional encapsulation area (*R*_e/ne_) (Fritz et al., 2017):

(3) $$R_{e/\mathrm{ne}}=\frac{A_{\text{encapsulated}}}{A_{\text{notencapsulated}}}$$

Furthermore, the area (mm^2^) of the encapsulation (*A*_E_) was determined (Fritz et al., 2017):

(4) $$A_{E}=A_{\text{encapsulated}}-A_{\text{notencapsulated}}$$

To determine the dry weight of the spiders and subsequently the PUFA profile of the individual spiders, they were lyophilized to complete dryness and weighed to the nearest 0.1 μg. Additionally, the basic food sources and chironomids were lyophilized to complete dryness. Individual chironomids were pooled and the pooled samples were weighed to the nearest 0.1 μg, For the basic food sources, approximately 40 mg with a precision of 0.1 μg were weighed. The PUFA profiles of adult chironomids and basic food sources were also analyzed. During the experiment, no samples were touched directly to avoid PUFA cross‐contamination from fingers to the samples. This was ensured by the use of tweezers, suction samplers, or gloves.

The PUFA of all samples were extracted based on Folch et al. (1957) with chloroform/methanol (5 mL, v:v; 2:1) over night at –20°C and the addition of an internal standard (C17:00200 μg mL^−1^; C23:0250 μg mL^−1^, Sigma‐Aldrich). After extraction, the samples were filtered through a syringe filter (PTFE, 13 mm, 0.45 μm, BGB) and evaporated until dryness under nitrogen at 40°C. Then, the samples were redissolved in methanol and stored until derivatization under nitrogen at −20°C. The PUFA in the samples were derivatized to fatty acid methyl esters (FAME) with methanolic trimethylsulfonium hydroxide (TMSH, 0.2 M, Macherey‐Nagel) at room temperature for 60 min. The FAMEs were analyzed using a gas chromatograph with a flame ionization detector (Varian CP‐3800, Varian Inc) equipped with a DB‐225 capillary column (30 m × 0.25 mm × 0.25 μm, Agilent J&W). External standards (Supelco 37 component FAME mix, 18:1n‐7 FAME, ALA FAME, Sigma‐Aldrich) were used to identify and quantify the FAMEs. The identification was made with OpenChrom version 1.4× (Wenig & Odermatt, 2010). Quantification was conducted in R version 4.2.0 (R Core Team, 2022). Details on the analytical procedure are given in the Appendix S1 under “Analytical procedure PUFA” and on the quantification in the Appendix S1 under “Calculation PUFA content.”

### 2.6 Data analysis

The spiders used at the beginning of the experiment for determining PUFA profiles, dry weight, and immune response were assigned randomly to the treatments to calculate the start values of the experiment. For the analysis, 11 PUFA (Figures S3, S4), commonly found in organisms, with ≥18 carbon atoms were used, including the physiological important PUFA ALA, LIN, EPA, and ARA. The content of a single PUFA was calculated as the proportion of total PUFA.

The PUFA profiles of spiders, chironomids, and basic food sources were visualized with nonmetric multidimensional scaling (NMDS) with Euclidean distances in two dimensions, which resulted in stress‐values <0.1. With analysis of similarity (ANOSIM; 999 permutations, Euclidean distance, *R*‐package vegan version 2.5–7, Oksanen et al., 2020), PUFA profiles of spiders were compared between time points within the same treatment and between treatments within the same time point. The latter was also done for chironomids and the basic food sources. To decrease the false discovery rate in multiple testing, *p*‐values were adjusted with the Benjamini–Hochberg method (Benjamini & Hochberg, 1995).

Whenever ANOSIM resulted in significant differences among treatments, similarity percentage (SIMPER) analyses (R‐package vegan version 2.5‐7, Oksanen et al. (2020)) were performed to identify PUFA contributing to the differences among treatments.

Fresh weight and body condition were analyzed with linear mixed models (LMM) including the explanatory variables time point and treatment as well as their interaction as fixed effects and replicate as random factor because the spider of a replicate was measured repeatedly. The LMM were fitted with the *R*‐package glmmTMB version 1.1.3 (Brooks et al., 2017). Linear models (LM) with time point, treatment, and their interaction as explanatory variables were used to analyze the dry weight and immune response, with the mean area of the encapsulation and proportional encapsulation area as response variables for the latter. No random factor was required for this analysis because spiders were measured only once for these responses. The effects of treatment, time point, and their interaction on the responses were tested by type II analysis of variance (ANOVA) using χ^2^‐test for the LMM and *F*‐tests for the LM. We removed the interaction term from the model when it was not statistically significant. All analyses were made with R version 4.2.0 (R Core Team, 2022). Including time point and treatment in the analysis covered the temporal dependence of the response variables. By testing for the treatment and time point effect in the same model, we were able to disentangle their effects. Whenever only time point was significant, the temporal dependence was stronger than the treatment effect. The data and R‐Scripts are openly available in GitHub at https://doi.org/10.5281/zenodo.7692685.

## 3 RESULTS

### 3.1 Change in PUFA profiles along the food chain

**FIGURE 2:**
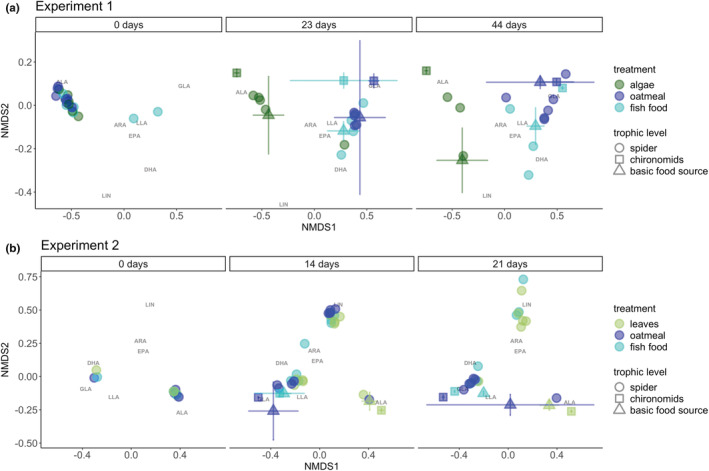
Nonmetric multidimensional scaling (NMDS) of the polyunsaturated fatty acid (PUFA) profiles in the first (a) and second (b) experiment. Colors indicate treatment, shape indicates trophic level. The first experiment included algae, fish food and oatmeal as food treatment, whereas the second experiment included leaves, fish food and oatmeal. For the chironomids and chironomid food sources mean and SD are presented. On Day 0, only PUFA profiles of spiders were analyzed to gain their starting values before they were fed with chironomids of the different treatments and the spiders were assigned randomly to the treatments. Chironomids and chironomid food sources were sampled and analyzed as soon as the spiders were fed with them. For a better overview only eicosapentaenoic acid (EPA, 20:5n‐3), arachidonic acid (ARA, 20:4n‐6), α‐linolenic acid (ALA, 18:3n‐3), ɣ‐linolenic acid (GLA, 18:3n‐6), linolelaidic acid (LLA, 18:2n‐6 t) and linoleic acid (LIN, 18:2n‐6) are displayed. The NMDS plot with all PUFA can be found in the Figure S2.

We found different PUFA profiles between treatments at all trophic levels of the food chain, except for spiders in the second experiment: In the first microcosm experiment, the PUFA profiles of spiders differed significantly across treatments for both treatment time points (ANOSIM: 23 days: *R* = 0.63, *p* = .010; 44 days: *R* = 0.69, *p* = .012). The spiders of the algae treatment were most strongly separated from the other treatments (Table 1 and Figure 2). In contrast, the spiders' PUFA profiles were similar for all treatments (ANOSIM: 14 days; *R* = −0.03, *p* = .693; 21 days: *R* = 0.21, *p* = .075) in the second experiment. The spiders had variable PUFA profiles at the beginning of each of the two experiments (Figure 2).

**TABLE 1:** Results of the analysis of similarities (ANOSIM) for the polyunsaturated fatty acids (PUFA) profiles.

Experiment	Compared groups			*R*	*p*‐Value
1	Treatments	Spiders	Time point 23 days	0.63	.010
			Time point 44 days	0.69	.012
		Chironomids	Time point 23 days	0.50	.012
			Time point 44 days	0.83	.006
		Basic food sources	Time point 23 days	0.71	.006
			Time point 44 days	0.68	.006
	Time points	Spiders	Treatment algae	0.11	.194
			Treatment oatmeal	0.80	.006
			Treatment fish food	0.38	.052
2	Treatments	Spiders	Time point 14 days	−0.03	.693
			Time point 21 days	0.21	.075
		Chironomids	Time point 14 days	0.94	.008
			Time point 21 days	1.00	.028
		Basic food sources	Time point 14 days	0.68	.008
			Time point 21 days	0.83	.030
	Time points	Spiders	Treatment leaves	0.26	.036
			Treatment oatmeal	0.14	.170
			Treatment fish food	0.30	.030

*Note*: Treatments were compared for spiders, chironomids, and basic food sources within the same time point and time points for spiders within the same treatment. *p*‐Values were adjusted using the Benjamini–Hochberg method. *R* values indicate the differences between groups: *R* < 0.25 barely separated, *R* < 0.5 clearly separated with some overlap, *R* > 0.75 well separated (Jaschinski et al., 2011).

In both experiments, the PUFA profiles of chironomids and the basic food sources differed statistically significant between the treatments at all time points (Table 1 and Figure 2). The treatments oatmeal and fish food were more similar to each other in both experiments then to the algae treatment in the first experiment and the leaves treatment in the second treatment (Figure 2).

### 3.2 Contribution of individual fatty acids to treatment differences

SIMPER analysis identified ALA, LIN, EPA, DHA, ɣ‐linolenic acid (GLA, 18:3n‐6), and linolelaidic acid (LLA, 18:2n‐6 t) as main contributors to the differences between treatments (SIMPER, Table 2, Figure 2). In both experiments, ALA and GLA contributed in most cases to the differences between treatments, while LIN, EPA, DHA and LLA explained the differences in only a few cases. In the first experiment, the entire algae treatment (algae, chironomids, and spiders) tended to contain higher levels of ALA than the other treatments (SIMPER, Table 2, Figure S3). For spiders between 39% and 43% of the differences in the PUFA profiles were explained by ALA, for chironomids between 49% and 50%, and for the basic food sources between 29% and 38%. The oatmeal treatment differed markedly from the algae and fish food treatments by the GLA content, except once the oatmeal treatments tended to contain more GLA than the other treatments (SIMPER, Table 2). In general, GLA explained between 27% and 45% of the differences in the PUFA profiles between the oatmeal and the other treatments. In the second experiment, the ALA content tended to be higher in chironomids of the leaves treatment than in the chironomids of the fish food and oatmeal treatment. Furthermore, leaves also tended to contain more ALA then fish food and oatmeal (SIMPER, Table 2, Figure S4). Overall, ALA explained between 23 and 49% of the differences between the treatments. The GLA content of the chironomids and base food sources differed markedly between the oatmeal and leaves as well as fish food treatments and explained between 31% and 49% of the differences in the PUFA profiles (SIMPER, Table 2).

**TABLE 2:** Results of the similarity percentage (SIMPER) analyses performed whenever analysis of similarity (ANOSIM) identified significant differences between treatments.

Experiment	Trophic level	Time point	Contrast	PUFA	Average	SD	Ratio	Average a	Average b	Cumulative contribution
1	Spiders	23 days	Algae and fish food	ALA	0.27	0.14	1.91	0.54	0	0.42
				GLA	0.24	0.11	2.28	0.09	0.57	0.79
			Algae and oatmeal	ALA	0.27	0.14	1.92	0.54	0	0.43
				GLA	0.26	0.09	2.69	0.09	0.6	0.83
			Fish food and oatmeal	GLA	0.05	0.03	1.48	0.57	0.6	0.27
				LLA	0.03	0.02	1.55	0.06	0.1	0.46
				EPA	0.03	0.02	1.33	0.14	0.16	0.63
				LIN	0.02	0.02	1.47	0.09	0.05	0.75
		44 days	Algae and fish food	ALA	0.24	0.09	2.69	0.58	0.1	0.39
				GLA	0.2	0.02	12.47	0	0.41	0.72
			Algae and oatmeal	GLA	0.33	0.07	4.38	0	0.65	0.45
				ALA	0.26	0.09	3.03	0.58	0.06	0.81
			Fish food and oatmeal	GLA	0.12	0.08	1.59	0.41	0.65	0.36
				ALA	0.06	0.08	0.81	0.1	0.06	0.55
				LLA	0.06	0.04	1.36	0.2	0.13	0.73
	Chironomids	23 days	Algae and fish food	ALA	0.38	0.17	2.18	0.97	0.22	0.49
				GLA	0.34	0.16	2.16	0	0.68	0.94
			Algae and oatmeal	ALA	0.48	0.02	27.31	0.97	0.01	0.50
				GLA	0.44	0.03	15.61	0	0.89	0.95
			Fish food and oatmeal	ALA	0.11	0.17	0.64	0.22	0.01	0.43
				GLA	0.11	0.16	0.68	0.68	0.89	0.83
		44 days	Algae and fish food	ALA	0.49	0.01	48.61	0.98	0	0.50
				GLA	0.43	0.01	42.46	0	0.86	0.93
			Algae and oatmeal	ALA	0.46	0.07	7.06	0.98	0.06	0.50
				GLA	0.42	0.05	7.98	0	0.83	0.94
			Fish food and oatmeal	GLA	0.03	0.04	0.76	0.86	0.83	0.31
				ALA	0.03	0.07	0.44	0	0.06	0.58
				EPA	0.02	0	4.23	0.04	0	0.77
1	Basic food sources	23 days	Algae and fish food	ALA	0.29	0.06	4.44	0.64	0.06	0.37
				GLA	0.19	0.08	2.35	0.12	0.5	0.61
				LIN	0.1	0.07	1.35	0.2	0.02	0.73
			Algae and oatmeal	GLA	0.31	0.13	2.31	0.12	0.72	0.38
				ALA	0.31	0.03	11.49	0.64	0.02	0.76
			Fish food and oatmeal	GLA	0.17	0.05	3.88	0.5	0.72	0.39
				DHA	0.08	0.01	6.23	0.17	0	0.57
				LIN	0.07	0.13	0.57	0.02	0.15	0.74
		44 days	Algae and fish food	ALA	0.24	0.11	2.22	0.52	0.07	0.29
				GLA	0.23	0.05	4.86	0.06	0.53	0.58
				LIN	0.15	0.05	3.05	0.35	0.05	0.77
			Algae and oatmeal	GLA	0.34	0.15	2.3	0.06	0.72	0.43
				ALA	0.24	0.11	2.19	0.52	0.17	0.73
			Fish food and oatmeal	GLA	0.18	0.04	4.54	0.53	0.72	0.39
				ALA	0.1	0.16	0.63	0.07	0.17	0.61
				DHA	0.06	0.03	2.15	0.13	0	0.74
2	Chironomids	14 days	Fish food and leaves	ALA	0.4	0.09	4.57	0.1	0.9	0.49

			GLA	0.35	0.08	4.44	0.71	0	0.92

		Fish food and oatmeal	GLA	0.1	0.08	1.22	0.71	0.9	0.45

			EPA	0.05	0.01	8.79	0.1	0	0.67

			ALA	0.05	0.09	0.56	0.1	0	0.90

		Leaves and oatmeal	GLA	0.45	0.01	83.97	0	0.9	0.48

			ALA	0.45	0.02	28.95	0.9	0	0.95

	21 days	Fish food and leaves	ALA	0.46	0.01	77.11	0	0.92	0.49

			GLA	0.4	0	910.85	0.8	0	0.93

		Fish food and oatmeal	GLA	0.07	0.01	7.77	0.8	0.94	0.43

			EPA	0.06	0	24.74	0.12	0	0.83

		Leaves and oatmeal	GLA	0.47	0.01	56.68	0	0.94	0.49

			ALA	0.46	0.01	78.92	0.92	0	0.96

basic food sources	14 days	Oatmeal and fish food	GLA	0.17	0.07	2.38	0.69	0.6	0.46

			LLA	0.11	0.09	1.24	0.26	0.25	0.77

		Oatmeal and leaves	ALA	0.36	0.03	11.52	0.04	0.77	0.42

			GLA	0.32	0.15	2.13	0.69	0.08	0.78

		Fish food and leaves	ALA	0.36	0.03	10.53	0.04	0.77	0.44

			GLA	0.26	0.05	5.6	0.6	0.08	0.75

	21 days	Fish food and leaves	ALA	0.28	0.03	8.58	0.16	0.72	0.46

			GLA	0.18	0.04	4.94	0.52	0.16	0.75

		Fish food and oatmeal	ALA	0.23	0.17	1.34	0.16	0.45	0.41

			GLA	0.21	0.05	3.98	0.52	0.43	0.81

		Leaves and oatmeal	ALA	0.23	0.14	1.56	0.72	0.45	0.43

			GLA	0.21	0.15	1.46	0.16	0.43	0.84

*Note*: Average is the contribution of polyunsaturated fatty acids (PUFA) to the average between‐group dissimilarity, ratio the average to SD ratio, average a the average abundance per first group in contrast and average b the average abundance per second group. Only PUFA with the closest higher cumulative contribution to 0.7 are displayed. EPA: eicosapentaenoic acid (20:5n‐3), ALA: α‐linolenic acid (18:3n‐3), GLA ɣ‐linolenic acid (18:3n‐6), LLA: linolelaidic acid (18:2n‐6 t), and LIN: linoleic acid (18:2n‐6).

### 3.3 Change in PUFA profiles over time

In the first experiment between time points, the PUFA profiles of spiders differed only in the oatmeal treatment (ANOSIM: *R* = 0.80, *p* = .006). In the second experiment, the spiders' PUFA profiles were similar for all treatments (ANOSIM: 14 days; *R* = −0.03, *p* = .693; 21 days: *R* = 0.21, *p* = .075), but differed significantly between time points of the leaves and fish food treatment (Table 1 and Figure 2).

### 3.4 Response of fresh and dry weight, growth rate, body condition, and immune response

**FIGURE 3:**
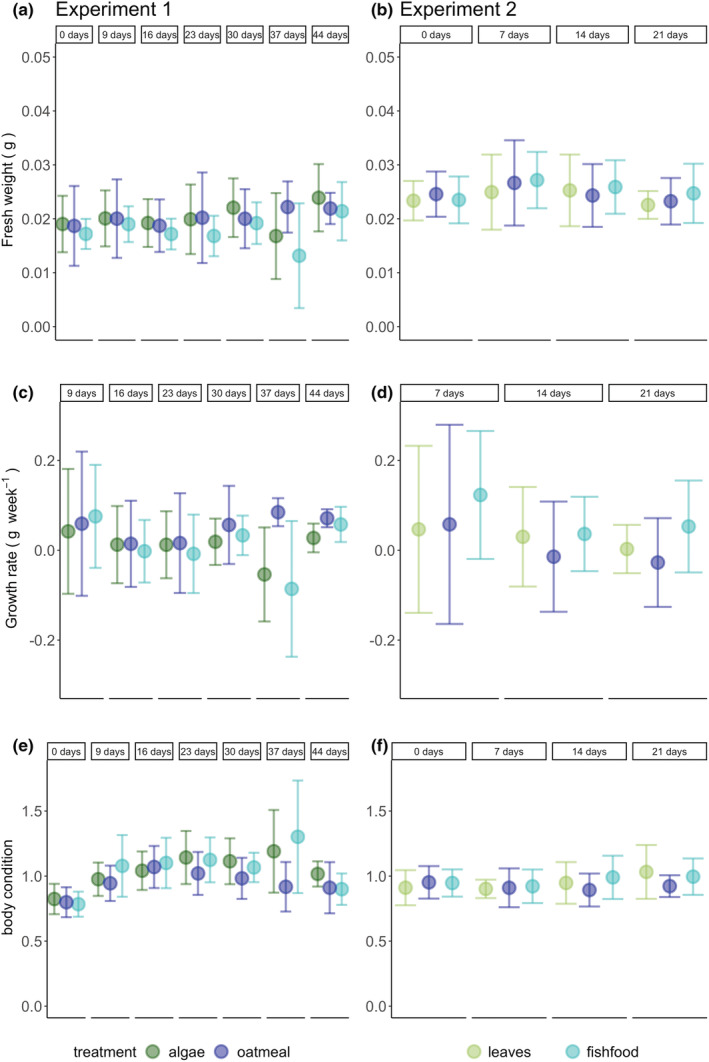
Mean and SD of fresh weight, growth rate and body condition of the spiders. (a, c) First experiment. (b, d) Second experiment. Colors indicate treatments: The first experiment included algae, fish food and oatmeal as food treatment, whereas the second experiment included leaves, fish food and oatmeal. In the first mesocosm experiment time point was significant for growth rate, time point and the interaction of treatment and time point for fresh weight and time point, treatment and their interaction for body condition. In the second experiment time point was significant for fresh weight and growth rate.

We aimed to identify a potential influence of dietary PUFA on the physiology and immune response of spiders. We found an effect of treatment only on fresh weight and body condition in the first experiment. The interaction between treatment and time point was significant (LMM, Table 3, Figure 3), whereas during the second experiment, only time point affected the fresh weight of spiders significantly (χ^2^ = 12.44, *p* = .006).

**TABLE 3:** Effects of the explanatory variables treatment and time point as well as their interaction on the fresh weight and body condition of spiders tested with type II analysis of variance (ANOVA) with χ^2^‐test for the linear mixed models (LMM).

Experiment	Response	Explanatory variable	df	χ^2^	*p*‐Value
1	**Fresh weight**	**Time point**	**6**	**27.66**	**.0001**
		Treatment	2	3.07	.2157
		**Treatment: time point**	**12**	**28.24**	**.0051**
	**Growth rate**	**Time point**	**5**	**30.74**	**<.0001**
		Treatment	2	0.38	.8272
	**Body condition**	**Time point**	**6**	**147.01**	**<.0001**
		**Treatment**	**2**	**6.95**	**.0310**
		**treatment: time point**	**12**	**24.30**	**.0185**
2	**Fresh weight**	**Time point**	**3**	**12.44**	**.0060**
		Treatment	2	0.50	.7780
	**Growth rate**	**Time point**	**2**	**6.83**	**.0329**
		Treatment	2	3.84	.1467
	Body condition	Time point	3	4.79	.1878
		Treatment	2	1.51	.4698

*Note*: Degrees of freedom (df), χ^2^ and *p*‐value of the ANOVA for fresh weight, growth rate, and body condition of the spiders. Statistical significance is indicated in bold. When the interaction of treatment and time point was not significant, the interaction was not included in the final model.

**FIGURE 4:**
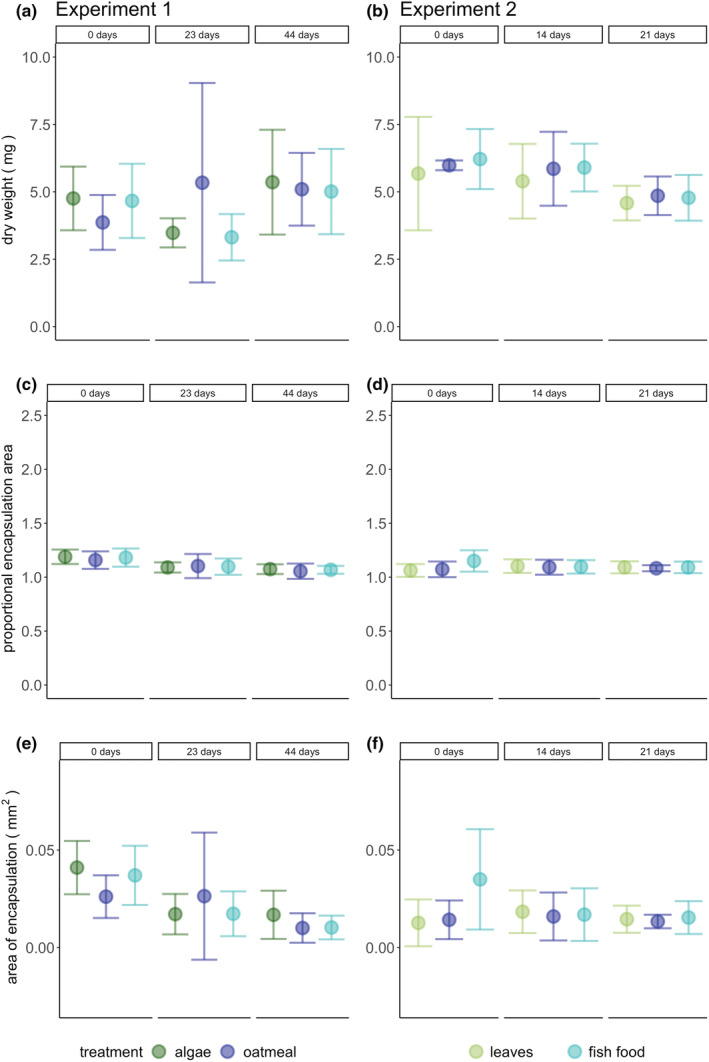
Mean and SD of dry weight, proportional encapsulation area and area of encapsulation of the spiders. (a, c, e) First experiment. (b, d, f) Second experiment. Colors indicate treatments: The first experiment included algae, fish food and oatmeal as food treatment, whereas the second experiment included leaves, fish food and oatmeal. In the first experiment time point was significant for proportional encapsulation area and area of encapsulation. In the second experiment time point was significant for dry weight.

No significant effects of treatment on the immune response and dry weight in the first and second experiment were found. During both experiments, the time point was significant for growth rate, but no directional trend was visible (LMM: first experiment: χ^2^ = 30.74, *p* < .001; second experiment: χ^2^ = 6.83, *p* = .033, Figure 3). Both, the proportional encapsulation area (LM: *F* = 8.86, *p* < .001) and the area of encapsulation (*F* = 7.33, *p* = 0.002) were reduced significantly with time in the first experiment (Figure 4), but not in the second experiment (Table 4). Conversely, dry weight was similar across treatment and time in the first experiment, but was reduced significantly with the time (LM: *F* = 5.71, *p* = .006) in the second experiment (Table 4).

**TABLE 4:** Effects of the explanatory variables treatment and time point on the dry weight, proportional encapsulation area and area of encapsulation of spiders tested with type II analysis of variance (ANOVA) with *F*‐test for the linear models (LM).

Experiment	Response	Explanatory variable	df	*F*	*p*‐Value
1	Dry weight	Time point	2	1.19	.3159
		Treatment	2	0.10	.9074
	**Proportional encapsulation area**	**Time point**	**2**	**8.86**	**.0008**
		Treatment	2	0.16	.8547
	**Area of encapsulation**	**Time point**	**2**	**7.33**	**.0023**
		Treatment	2	0.37	.6906
2	**Dry weight**	**Time point**	**2**	**5.71**	**.0056**
		Treatment	2	0.86	.4285
	Proportional encapsulation area	Time point	2	0.11	.8929
		Treatment	2	0.55	.5778
	Area of encapsulation	Time point	2	0.78	.4614
		Treatment	2	1.04	.3614

*Note*: Degrees of freedom (df), *F* and *p*‐value of the ANOVA for dry weight and immune response of the spiders. Statistical significance is indicated in bold. The interaction of treatment and time point was not significant. Therefore, the interaction was not included in the final models.

## 4 DISCUSSION

### 4.1 Change in PUFA profiles along the food chain

We aimed to identify the influence of dietary PUFA on PUFA profiles along a tritrophic food chain with different basic food sources. As expected, we found differences in PUFA profiles between treatments at all trophic levels of the food chain, except for the spiders in the second experiment. Temperature in the second experiment was higher than in the first experiment (Figure S1) and may explain the absence of the treatment effect. Temperature is known to reduce the PUFA content of aquatic organisms as a response to decrease the fluidity of their cell membranes (Arts & Kohler, 2009; Fuschino et al., 2011).

Furthermore, spiders showed high mortality across the experiments, which suggests that the spiders including those surviving were stressed. The stress may have been caused by differences such as the absence of a forested stream in the microcosms compared with their natural habitats resulting in less humidity and shading as well as higher temperatures. Stress requires to invest more energy for maintenance (Calow & Forbes, 1998; Sokolova et al., 2012), thereby reducing the energy available for energetically costly biosynthesis of PUFA (Mathieu‐Resuge et al., 2022; Twining, Bernhardt, et al., 2021; Twining, Parmar, et al., 2021) and other physiological processes such as growth (Calow & Forbes, 1998; Sokolova et al., 2012).

In the first experiment, the PUFA profiles of spiders were more similar to the basic food sources than to the chironomids (Figure 2). Other factors than dietary PUFA uptake can influence the PUFA profiles of spiders. One example is GLA, which was not found in chironomids fed with algae, but in spiders in the algae‐based treatment (Figure S3). The spiders may have stored GLA in their tissue or synthesized it from its precursor LIN (Horrobin, 1992). The latter has not been shown in spiders, but a recent study suggests that spiders are capable of synthesizing EPA from dietary C18 PUFA precursors (Mathieu‐Resuge et al., 2022). Further studies with compound‐specific stable carbon and hydrogen isotopes could help to track the trophic transfer of GLA and thereby improve understanding the effects of PUFA on organisms and in turn on food webs (Mathieu‐Resuge et al., 2022).

### 4.2 Contribution of individual fatty acids to treatment differences

The PUFA ALA and GLA and the PUFA LIN, EPA, DHA, and LLA were major and minor contributors to the differences between treatments, respectively. In contrast, to another study, which found no GLA in oats (Goedkoop et al., 2007), we found on trend higher GLA levels in oatmeal than in fish food, algae, and leaves. Additionally, the chironomids of the oatmeal‐based treatment tended also to contain higher GLA levels then the chironomids of the fish food‐, algae‐, and leave‐based treatment. Therefore, GLA levels in chironomids probably reflect the GLA levels of their diet, which is in line with Strandberg et al. (2020). In the first experiment, spiders of the oatmeal‐based treatment also exhibited higher GLA levels than the algae‐ and fish food‐based treatments. This suggests that the spiders GLA levels reflect their diet, though biosynthesis may also play a role as discussed above for the algae‐based treatment.

The algae and conditioned leaves had higher levels of ALA than the basic food sources fish food and oatmeal. This is in line with other studies that found ALA in higher amounts in algae than in fish food and oatmeal (Strandberg et al., 2020; Torres‐Ruiz et al., 2010). Furthermore, chironomids that consumed algae and leaves had higher levels of ALA than chironomids fed with oatmeal and fish food. Thus, ALA levels are likely driven by uptake from food sources, which is supported by studies showing that chironomid larvae (Strandberg et al., 2020) and caddisfly larvae (Torres‐Ruiz et al., 2010) rely on dietary ALA supply.

Similarly, spiders in the algae‐based food treatment displayed higher ALA levels than spiders of the oatmeal‐ and fish food‐based treatments, but the leave‐based food treatment did not show different ALA levels. Therefore, nondietary factors may affect the ALA levels in spiders. One factor can be the synthesis of EPA from ALA in spiders (Mathieu‐Resuge et al., 2022). Another factor can be temperature, which was higher during the second experiment with the leaves treatment (Figure S1). Temperature is known to affect PUFA profiles of invertebrates, as mentioned above. Specifically, ALA levels were shown to decrease with increasing temperature in *Daphnia magna* (Martin‐Creuzburg et al., 2019; Zeis et al., 2019) and terrestrial vertebrates (Hagve et al., 1998; Lund et al., 1999), whereas studies on terrestrial invertebrates, including spiders, are lacking. Additionally, higher temperatures increase the metabolic rate and in turn energy demand of organisms (Brown et al., 2004). As ALA is an energy source for organisms (Tocher, 2003), the spiders may have used ALA to meet their energy demand, thereby decreasing ALA levels.

Anthropogenic stressors such increasing temperatures can have direct effects on the PUFA profiles of organisms, for example, temperature was shown to reduce long‐chain PUFA in algae (Hixson & Arts, 2016) and to cause indirect effects by altering species assemblages (Hixson et al., 2015; Martin‐Creuzburg et al., 2017) and in turn the availability of PUFA for consumers. Given the wide occurrence of multiple anthropogenic stressors, a realistic assessment for most environmental conditions requires studies that consider the effects of anthropogenic stressors on PUFA transfer and organisms (Kowarik et al., 2022). This is also important in light of vertebrates with a high conservation status, such as birds and bats, potentially relying on long‐chain PUFA in spiders. These aspects need also to be taken into account to estimate PUFA transfer from aquatic to terrestrial ecosystems.

### 4.3 Change in PUFA profiles over time

As expected, we found temporal changes in PUFA profiles of the spiders during the experiments. In both experiments, the spiders showed a high variability in initial PUFA profiles, for example in the second experiment two distinct groups were present (Figure 2). This variability may be due to different food sources consumed by the spiders in the field prior to collection. The initial PUFA profiles of the spiders could have affected the PUFA profiles of the spiders during the experiment and thereby the response to treatments (Galloway & Budge, 2020). For example, the initial EPA levels of spiders were similar to the EPA levels later in the experiment. The spiders were collected near streams, so their body may have stored EPA originating from aquatic emergent insects that contain EPA in relatively high amounts (Hixson et al., 2015; Martin‐Creuzburg et al., 2017; Moyo et al., 2017). Furthermore, spiders are theoretically capable of synthesizing EPA (Mathieu‐Resuge et al., 2022), which may not occur when the spiders retained EPA from their diet prior to collection (Galloway & Budge, 2020). Hence, a sufficiently long experimental duration is required to minimize the effect of the initial PUFA profiles and to detect treatment effects. We addressed this issue by analyzing the PUFA profiles at different timepoints, but the absence of treatment effects on the PUFA profiles of spiders in the second experiment may be explained by an insufficiently long experimental duration owing to the higher mortality.

### 4.4 Response of spiders fresh and dry weight, growth rate, and body condition to the basic food treatments

We aimed to identify a potential influence of dietary PUFA on the physiology of spiders. We found a treatment effect only in the first experiment, where the interaction of treatment and time point was significant for fresh weight and body condition of spiders, while no effect on dry weight was found. The significant interaction shows that the effect of the treatment on body condition and fresh weight depends on the time point. The PUFA profiles of spiders differed also significantly between treatments. This is in line with Mayntz and Toft (2001), who showed that PUFA enhanced fresh weight of spiders.

By contrast, in the second experiment, fresh weight and dry weight differed between time points but not between treatments, while body condition was similar across treatments and time points. Furthermore, the PUFA profiles of the spiders were similar across treatments in the second experiment. The different response of spiders across the experiments may be explained by the study durations and conditions such as temperature. The first experiment lasted for approximately 6 weeks, whereas the second microcosm experiment was terminated after 3 weeks. A longer study duration may have resulted in detectable treatment effects also in the second experiment. Furthermore, the temperature during the second experiment was higher (Figure S1), and it was shown that with increasing temperature, the requirements of organisms for PUFA can be reduced (Masclaux et al., 2009): Growth and reproduction rates decreased in a study on zooplankton with decreasing PUFA quality, though the PUFA effect was reduced at higher temperature and became negligible. Similar results were found for *D. magna*, whose growth generally increased with increasing temperature, but a significant positive effect of PUFA‐rich diets on growth was only observed at the lowest temperature of 10°C (Martin‐Creuzburg et al., 2012). In contrast, at higher temperatures, a greater PUFA content in *D. magna* may even enhance the vulnerability to heat‐induced oxidative stress (Zeis et al., 2019), because the double bonds in PUFA are prone to oxidation by reactive oxygen species. Furthermore, the heat tolerance of *D. magna* can be reduced with increasing PUFA uptake (Martin‐Creuzburg et al., 2019). Therefore, beneficial effects of dietary PUFA intake for spiders may be counterbalanced by increased oxidative stress and reduced heat tolerance at higher temperatures. Thus, other factors such as temperature may have been more important than dietary PUFA intake for growth rate, dry weight, and body condition.

### 4.5 Immune response of spiders to the basic food treatments

Contrary to our expectation, the immune response of spiders was only affected significantly by time point and not by treatment. This is in contrast to a field study, in which the long‐chain PUFA EPA and DHA were linked to enhanced immune responses of spiders (Fritz et al., 2017). In our study, food sources of spiders contained only low levels of these long‐chain PUFA, which may explain the absence of a treatment effect. Nonetheless, the immune response is affected by many factors not considered in our experiments, which may explain our finding. For instance, the immune response can decrease with reduced food intake (Siva‐Jothy & Thompson, 2002) and changes in the dietary composition (Srygley et al., 2009). In our study, spiders received only one food source. In real‐world ecosystems, spiders consume and benefit from multiple prey types (Nyffeler, 1999; Uetz et al., 1992). That is because a balanced nutrient composition of prey is more important for the performance, for example, survival of spiders, than single nutrients (Mayntz & Toft, 2001). Additionally, depending on their hunting strategy, spiders are capable to obtain an optimal nutrient composition through adjusting foraging strategies. For example, mobile spiders such as ground hunters are capable to choose prey actively (Mayntz et al., 2005). Furthermore, population density (Schmid‐Hempel, 2005; Wilson & Cotter, 2008), environmental factors (Adamo, 2012; Wojda, 2017), and anthropogenic stressors (Mangahas et al., 2019) can affect the immune response. Future studies that also consider other factors such as nutrient availability and anthropogenic stressors can help to estimate the importance of PUFA for immune response in relation to other factors.

## 5 CONCLUSIONS

PUFA can affect the weight and body condition of spiders, where this depends on the environmental context. This context includes, among others, diverse food sources, that is, several prey types, food chains with interactions between taxa, long duration for PUFA assimilation, a range of environmental factors (e.g., temperature), and anthropogenic stressors.

Furthermore, the PUFA profiles can differ across trophic levels for multiple food sources. Aquatic and semi‐aquatic food sources may result in more distinct PUFA profiles of chironomids and spiders than terrestrial food sources. ALA and GLA are among the major contributors to these differences in PUFA between food sources. However, environmental factors such as temperature also influence PUFA profiles. Future studies under more realistic conditions are needed to improve our understanding of the effect of PUFA in ecosystems and to evaluate the transferability of our results.

## AUTHOR CONTRIBUTIONS

**Katharina Ohler:** Conceptualization (equal); data curation (equal); formal analysis (equal); investigation (equal); methodology (equal); project administration (equal); visualization (equal); writing – original draft (lead); writing – review and editing (equal). **Verena C. Schreiner:** Formal analysis (equal); methodology (equal); visualization (equal); writing – review and editing (equal). **Dominik Martin Creuzburg:** Methodology (equal); visualization (equal); writing – review and editing (equal). **Ralf B. Schfer:** Conceptualization (equal); formal analysis (equal); methodology (equal); project administration (equal); supervision (equal); visualization (equal); writing – original draft (supporting); writing – review and editing (equal).

## FUNDING INFORMATION

This study was supported by the German Research Foundation (DFG, Project Number 326210499/GRK 2360). Katharina Ohler was funded by the German Academic Scholarship Foundation and the Interdisziplinäres Promotions‐ und Postdoczentrum (IPZ) completion scholarship.

## CONFLICT OF INTEREST STATEMENT

The authors declare no conflict of interest.

### OPEN RESEARCH BADGES

This article has earned an Open Data badge for making publicly available the digitally‐shareable data necessary to reproduce the reported results. The data is available at https://doi.org/10.5281/zenodo.7692685.

## Supporting information


Appendix S1

## Data Availability

The data that support the findings of this study are openly available in GitHub at https://doi.org/10.5281/zenodo.7692685

## References

[ece39927-bib-0001] Adamo, S. A. (2012). The effects of the stress response on immune function in invertebrates: An evolutionary perspective on an ancient connection. Hormones and Behavior, 62, 324–330. 10.1016/j.yhbeh.2012.02.012 22381405

[ece39927-bib-0002] Ahlgren, G. , Gustafsson, I.‐B. , & Boberg, M. (1992). Fatty acid content and chemical composition of freshwater microalgae 1. Journal of Phycology, 28, 37–50. 10.1111/j.0022-3646.1992.00037.x

[ece39927-bib-0003] Ahtiainen, J. J. , Alatalo, R. V. , Kortet, R. , & Rantala, M. J. (2004). Sexual advertisement and immune function in an arachnid species (Lycosidae). Behavioral Ecology, 15, 602–606. 10.1093/beheco/arh062

[ece39927-bib-0004] Ahtiainen, J. J. , Alatalo, R. V. , Kortet, R. , & Rantala, M. J. (2005). A trade‐off between sexual signalling and immune function in a natural population of the drumming wolf spider *Hygrolycosa rubrofasciata* . Journal of Evolutionary Biology, 18, 985–991. 10.1111/j.1420-9101.2005.00907.x 16033571

[ece39927-bib-0005] Anderson, J. F. (1974). Responses to starvation in the spiders Lycosa Lenta Hentz and Filistata Hibernalis (Hentz). Ecology, 55, 576–585. 10.2307/1935148

[ece39927-bib-0006] Arts, M. T. , Ackman, R. G. , & Holub, B. J. (2001). “Essential fatty acids” in aquatic ecosystems: A crucial link between diet and human health and evolution. Canadian Journal of Fisheries and Aquatic Sciences, 58, 122–137. 10.1139/f00-224

[ece39927-bib-0007] Arts, M. T. , & Kohler, C. C. (2009). Health and condition in fish: The influence of lipids on membrane competency and immune response. In M. Kainz , M. T. Brett , & M. T. Arts (Eds.), Lipids in aquatic ecosystems (pp. 237–256). Springer. 10.1007/978-0-387-89366-2_10

[ece39927-bib-0008] Bärlocher, F. (1985). The role of fungi in the nutrition of stream invertebrates. Botanical Journal of the Linnean Society, 91, 83–94. 10.1111/j.1095-8339.1985.tb01137.x

[ece39927-bib-0009] Baxter, C. V. , Fausch, K. D. , & Saunders, W. C. (2005). Tangled webs: Reciprocal flows of invertebrate prey link streams and riparian zones. Freshwater Biology, 50, 201–220. 10.1111/j.1365-2427.2004.01328.x

[ece39927-bib-0010] Benjamini, Y. , & Hochberg, Y. (1995). Controlling the false discovery rate: A practical and powerful approach to multiple testing. Journal of the Royal Statistical Society: Series B: Methodological, 57, 289–300. 10.1111/j.2517-6161.1995.tb02031.x

[ece39927-bib-0011] Brett, M. , & Müller‐Navarra, D. (1997). The role of highly unsaturated fatty acids in aquatic foodweb processes. Freshwater Biology, 38, 483–499. 10.1046/j.1365-2427.1997.00220.x

[ece39927-bib-0012] Brooks, M. E. , Kristensen, K. , van Benthem, K. J. , Magnusson, A. , Berg, C. W. , Nielsen, A. , Skaug, H. J. , Mächler, M. , & Bolker, B. M. (2017). glmmTMB balances speed and flexibility among packages for zero‐inflated generalized linear mixed modeling. R Journal, 9, 378–400.

[ece39927-bib-0013] Brown, J. H. , Gillooly, J. F. , Allen, A. P. , Savage, V. M. , & West, G. B. (2004). Toward a metabolic theory of ecology. Ecology, 85, 1771–1789. 10.1890/03-9000

[ece39927-bib-0014] Calow, P. , & Forbes, V. E. (1998). How do physiological responses to stress translate into ecological and evolutionary processes? Comparative Biochemistry and Physiology. Part A, Molecular & Integrative Physiology, 120, 11–16. 10.1016/S1095-6433(98)10003-X

[ece39927-bib-0015] Davis, B. N. K. (1989). The European distribution of insects on stinging nettles, *Urtica dioica* L.: A field survey. Bollettino di Zoologia, 56, 321–326. 10.1080/11250008909355658

[ece39927-bib-0016] Foelix, R. F. (2011). Biology of spiders (3rd ed.). Oxford University Press.

[ece39927-bib-0017] Folch, J. , Lees, M. , & Stanley, G. H. S. (1957). A simple method for the isolation and purification of total LIPIDES from animal tissues. The Journal of Biological Chemistry, 226, 497–509. 10.1016/S0021-9258(18)64849-5 13428781

[ece39927-bib-0018] Fritz, K. A. , Kirschman, L. J. , McCay, S. D. , Trushenski, J. T. , Warne, R. W. , & Whiles, M. R. (2017). Subsidies of essential nutrients from aquatic environments correlate with immune function in terrestrial consumers. Freshwater Science, 36, 893–900. 10.1086/694451

[ece39927-bib-0019] Fuschino, J. R. , Guschina, I. A. , Dobson, G. , Yan, N. D. , Harwood, J. L. , & Arts, M. T. (2011). Rising water temperatures Alter lipid dynamics and reduce N‐3 essential fatty acid concentrations in *Scenedesmus obliquus* (chlorophyta)1. Journal of Phycology, 47, 763–774. 10.1111/j.1529-8817.2011.01024.x 27020012

[ece39927-bib-0020] Galloway, A. W. E. , & Budge, S. M. (2020). The critical importance of experimentation in biomarker‐based trophic ecology. Philosophical Transactions of the Royal Society B, 375, 20190638. 10.1098/rstb.2019.0638 PMC7333966 32536303

[ece39927-bib-0021] Gladyshev, M. I. , Sushchik, N. N. , & Makhutova, O. N. (2013). Production of EPA and DHA in aquatic ecosystems and their transfer to the land. Prostaglandins other lipid mediator, 4th European Workshop on Lipid Mediators, 107, 117–126. 10.1016/j.prostaglandins.2013.03.002 23500063

[ece39927-bib-0022] Goedkoop, W. , Demandt, M. , & Ahlgren, G. (2007). Interactions between food quantity and quality (long‐chain polyunsaturated fatty acid concentrations) effects on growth and development of *Chironomus riparius* . Canadian Journal of Fisheries and Aquatic Sciences, 64, 425–436. 10.1139/f07-016

[ece39927-bib-0023] Graça, M. , & Canhoto, C. (2006). Leaf litter processing in low order streams. Limnetica, 25, 1–10.

[ece39927-bib-0024] Hagve, T.‐A. , Woldseth, B. , Brox, J. , Narce, M. , & Poisson, J.‐P. (1998). Membrane fluidity and fatty acid metabolism in kidney cells from rats fed purified eicosapentaenoic acid or purified docosahexaenoic acid. Scandinavian Journal of Clinical and Laboratory Investigation, 58, 187–194. 10.1080/00365519850186571 9670342

[ece39927-bib-0025] Henschel, J. R. , Mahsberg, D. , & Stumpf, H. (2001). Allochthonous aquatic insects increase predation and decrease herbivory in river shore food webs. Oikos, 93, 429–438. 10.1034/j.1600-0706.2001.930308.x

[ece39927-bib-0026] Hixson, S. M. , & Arts, M. T. (2016). Climate warming is predicted to reduce omega‐3, long‐chain, polyunsaturated fatty acid production in phytoplankton. Global Change Biology, 22, 2744–2755. 10.1111/gcb.13295 27070119

[ece39927-bib-0027] Hixson, S. M. , Sharma, B. , Kainz, M. J. , Wacker, A. , & Arts, M. T. (2015). Production, distribution, and abundance of long‐chain omega‐3 polyunsaturated fatty acids: A fundamental dichotomy between freshwater and terrestrial ecosystems. Environmental Reviews, 23, 414–424. 10.1139/er-2015-0029

[ece39927-bib-0028] Horrobin, D. F. (1992). Nutritional and medical importance of gamma‐linolenic acid. Progress in Lipid Research, 31, 163–194. 10.1016/0163-7827(92)90008-7 1334266

[ece39927-bib-0029] Jakob, E. M. , Marshall, S. D. , & Uetz, G. W. (1996). Estimating fitness: A comparison of body condition indices. Oikos, 77, 61–67. 10.2307/3545585

[ece39927-bib-0030] Jaschinski, S. , Brepohl, D. C. , & Sommer, U. (2011). Seasonal variation in carbon sources of mesograzers and small predators in an eelgrass community: Stable isotope and fatty acid analyses. Marine Ecology Progress Series, 431, 69–82. 10.3354/meps09143

[ece39927-bib-0031] Kainz, M. , Arts, M. T. , & Mazumder, A. (2004). Essential fatty acids in the planktonic food web and their ecological role for higher trophic levels. Limnology and Oceanography, 49, 1784–1793. 10.4319/lo.2004.49.5.1784

[ece39927-bib-0032] Kato, C. , Iwata, T. , & Wada, E. (2004). Prey use by web‐building spiders: Stable isotope analyses of trophic flow at a forest‐stream ecotone. Ecological Research, 19, 633–643. 10.1111/j.1440-1703.2004.00678.x

[ece39927-bib-0033] Kowarik, C. , Martin‐Creuzburg, D. , Mathers, K. L. , Weber, C. , & Robinson, C. T. (2022). Stream degradation affects aquatic resource subsidies to riparian ground‐dwelling spiders. Science Total Environment, 855, 158658. 10.1016/j.scitotenv.2022.158658 36113799

[ece39927-bib-0034] Kowarik, C. , Martin‐Creuzburg, D. , & Robinson, C. T. (2021). Cross‐ecosystem linkages: Transfer of polyunsaturated fatty acids from streams to riparian spiders via emergent insects. Frontiers in Ecology and Evolution, 9, 707570. 10.3389/fevo.2021.707570

[ece39927-bib-0035] Lund, E. K. , Harvey, L. J. , Ladha, S. , Clark, D. C. , & Johnson, I. T. (1999). Effects of dietary fish oil supplementation on the phospholipid composition and fluidity of cell membranes from human volunteers. Annals of Nutrition & Metabolism, 43, 290–300. 10.1159/000012797 10749029

[ece39927-bib-0036] Mangahas, R. S. , Murray, R. L. , & McCauley, S. J. (2019). Chronic exposure to high concentrations of road salt decreases the immune response of dragonfly larvae. Frontiers in Ecology and Evolution, 7, 376.

[ece39927-bib-0037] Marcarelli, A. M. , Baxter, C. V. , Mineau, M. M. , & Hall, R. O. (2011). Quantity and quality: Unifying food web and ecosystem perspectives on the role of resource subsidies in freshwaters. Ecology, 92, 1215–1225. 10.1890/10-2240.1 21797150

[ece39927-bib-0038] Martin‐Creuzburg, D. , Coggins, B. L. , Ebert, D. , & Yampolsky, L. Y. (2019). Rearing temperature and fatty acid supplementation jointly affect lipid fluorescence polarization and heat tolerance in daphnia. Physiological and Biochemical Zoology, 92, 408–418. 10.1086/704365 31180800

[ece39927-bib-0039] Martin‐Creuzburg, D. , Kowarik, C. , & Straile, D. (2017). Cross‐ecosystem fluxes: Export of polyunsaturated fatty acids from aquatic to terrestrial ecosystems via emerging insects. Science Total Environment, 577, 174–182. 10.1016/j.scitotenv.2016.10.156 27810302

[ece39927-bib-0040] Martin‐Creuzburg, D. , Wacker, A. , Ziese, C. , & Kainz, M. J. (2012). Dietary lipid quality affects temperature‐mediated reaction norms of a freshwater key herbivore. Oecologia, 168, 901–912. 10.1007/s00442-011-2155-1 22002040

[ece39927-bib-0041] Masclaux, H. , Bec, A. , Kainz, M. J. , Desvilettes, C. , Jouve, L. , & Bourdier, G. (2009). Combined effects of food quality and temperature on somatic growth and reproduction of two freshwater cladocerans. Limnology and Oceanography, 54, 1323–1332. 10.4319/lo.2009.54.4.1323

[ece39927-bib-0042] Mathieu‐Resuge, M. , Pilecky, M. , Twining, C. W. , Martin‐Creuzburg, D. , Parmar, T. P. , Vitecek, S. , & Kainz, M. J. (2022). Dietary availability determines metabolic conversion of long‐chain polyunsaturated fatty acids in spiders: A dual compound‐specific stable isotope approach. Oikos, 2022(7). 10.1111/oik.08513

[ece39927-bib-0043] Mayntz, D. , Raubenheimer, D. , Salomon, M. , Toft, S. , & Simpson, S. J. (2005). Nutrient‐specific foraging in invertebrate predators. Science, 307, 111–113. 10.1126/science.1105493 15637278

[ece39927-bib-0044] Mayntz, D. , & Toft, S. (2001). Nutrient composition of the prey's diet affects growth and survivorship of a generalist predator. Oecologia, 127, 207–213. 10.1007/s004420000591 24577651

[ece39927-bib-0045] Moyo, S. , Chari, L. D. , Villet, M. H. , & Richoux, N. B. (2017). Decoupled reciprocal subsidies of biomass and fatty acids in fluxes of invertebrates between a temperate river and the adjacent land. Aquatic Sciences, 79, 689–703. 10.1007/s00027-017-0529-0

[ece39927-bib-0046] Nakano, S. , & Murakami, M. (2001). Reciprocal subsidies: Dynamic interdependence between terrestrial and aquatic food webs. Proceedings of the National Academy of Sciences, 98, 166–170. 10.1073/pnas.98.1.166 PMC14562 11136253

[ece39927-bib-0047] Nyffeler, M. (1999). Prey selection of spiders in the field. Journal of Arachnology, 27, 317–324.

[ece39927-bib-0048] OECD . (2011). Guideline for the testing of chemicals 235: Chironomus sp., acute immobilisation test. OECD.

[ece39927-bib-0049] Oksanen, J. , Blanchet, F. G. , Friendly, M. , Kindt, R. , Legendre, P. , McGlinn, D. , Minchin, P. , O'Hara, R. , Simpson, G. , Solymos, P. , Stevens, M. , Szöcs, E. , & Wagner, H. (2020). Vegan community ecology package version 2.5‐7 November 2020. https://CRAN.R‐project.org/package=vegan

[ece39927-bib-0050] Paetzold, A. , Smith, M. , Warren, P. H. , & Maltby, L. (2011). Environmental impact propagated by cross‐system subsidy: Chronic stream pollution controls riparian spider populations. Ecology, 92, 1711–1716. 10.1890/10-2184.1 21939066

[ece39927-bib-0051] Parmar, T. P. , Kindinger, A. L. , Mathieu‐Resuge, M. , Twining, C. W. , Shipley, J. R. , Kainz, M. J. , & Martin‐Creuzburg, D. (2022). Fatty acid composition differs between emergent aquatic and terrestrial insects: A detailed single system approach. Frontiers in Ecology and Evolution, 10, 814.

[ece39927-bib-0052] Poulin, B. , Lefebvre, G. , & Paz, L. (2010). Red flag for green spray: Adverse trophic effects of Bti on breeding birds. Journal of Applied Ecology, 47, 884–889. 10.1111/j.1365-2664.2010.01821.x

[ece39927-bib-0053] R Core Team . (2022). R: A language and environment for statistical computing. R Foundation for Statistical Computing.

[ece39927-bib-0054] Rantala, M. J. , Jokinen, I. , Kortet, R. , Vainikka, A. , & Suhonen, J. (2002). Do pheromones reveal male Immunocompetence? Proceedings of the National Academy of Sciences, India Section B, 269, 1681–1685. 10.1098/rspb.2002.2056 PMC1691089 12204128

[ece39927-bib-0055] Rasband, W. S. (2018). ImageJ. http://imagej.nih.gov/ij

[ece39927-bib-0056] Ratcliffe, N. A. , & Rowley, A. F. (1979). Role of hemocytes in defense against biological agents. In A. P. Gupta (Ed.), Insect Hemocytes: Development, forms, functions and techniques (pp. 331–414). Cambridge University Press. 10.1017/CBO9780511759987.014

[ece39927-bib-0057] Reitze, M. , & Nentwig, W. (1991). Comparative investigations into the feeding ecology of six Mantodea species. Oecologia, 86, 568–574. 10.1007/BF00318324 28313339

[ece39927-bib-0058] Sabo, J. L. , & Power, M. E. (2002). Numerical response of lizards to aquatic insects and short‐term consequences for terrestrial prey. Ecology, 83, 3023–3036.

[ece39927-bib-0059] Sayanova, O. V. , & Napier, J. A. (2004). Eicosapentaenoic acid: Biosynthetic routes and the potential for synthesis in transgenic plants. Phytochemistry, 65, 147–158. 10.1016/j.phytochem.2003.10.017 14732274

[ece39927-bib-0060] Scharnweber, K. , Chaguaceda, F. , Dalman, E. , Tranvik, L. , & Eklöv, P. (2020). The emergence of fatty acids—Aquatic insects as vectors along a productivity gradient. Freshwater Biology, 65, 565–578. 10.1111/fwb.13454

[ece39927-bib-0061] Schindler, D. E. , & Smits, A. P. (2017). Subsidies of aquatic resources in terrestrial ecosystems. Ecosystems, 20, 78–93. 10.1007/s10021-016-0050-7

[ece39927-bib-0062] Schmid‐Hempel, P. (2005). Evolutionary ecology of insect immune defenses. Annual Review of Entomology, 50, 529–551. 10.1146/annurev.ento.50.071803.130420 15471530

[ece39927-bib-0063] Siva‐Jothy, M. T. , & Thompson, J. J. W. (2002). Short‐term nutrient deprivation affects immune function. Physiological Entomology, 27, 206–212. 10.1046/j.1365-3032.2002.00286.x

[ece39927-bib-0064] Sokolova, I. M. , Frederich, M. , Bagwe, R. , Lannig, G. , & Sukhotin, A. A. (2012). Energy homeostasis as an integrative tool for assessing limits of environmental stress tolerance in aquatic invertebrates. Marine Environmental Research, 79, 1–15. 10.1016/j.marenvres.2012.04.003 22622075

[ece39927-bib-0065] Srygley, R. B. , Lorch, P. D. , Simpson, S. J. , & Sword, G. A. (2009). Immediate protein dietary effects on movement and the generalised immunocompetence of migrating Mormon crickets *Anabrus simplex* (Orthoptera: Tettigoniidae). Ecological Entomology, 34, 663–668. 10.1111/j.1365-2311.2009.01117.x

[ece39927-bib-0066] Strandberg, U. , Hiltunen, M. , Jelkänen, E. , Taipale, S. J. , Kainz, M. J. , Brett, M. T. , & Kankaala, P. (2015). Selective transfer of polyunsaturated fatty acids from phytoplankton to planktivorous fish in large boreal lakes. Science Total Environment, 536, 858–865. 10.1016/j.scitotenv.2015.07.010 26282609

[ece39927-bib-0067] Strandberg, U. , Vesterinen, J. , Ilo, T. , Akkanen, J. , Melanen, M. , & Kankaala, P. (2020). Fatty acid metabolism and modifications in *Chironomus riparius* . Philosophical Transactions of the Royal Society B, 375, 20190643. 10.1098/rstb.2019.0643 PMC7333955 32536306

[ece39927-bib-0068] Sullivan, C. M. , Shiel, C. B. , McAney, C. M. , & Fairley, J. S. (1993). Analysis of the diets of Leisler's Nyctalus leisleri, Daubenton's *Myotis daubentoni* and pipistrelle *Pipistrellus pipistrellus* bats in Ireland. Journal of Zoology, 231, 656–663. 10.1111/j.1469-7998.1993.tb01947.x

[ece39927-bib-0069] Tocher, D. R. (2003). Metabolism and functions of lipids and fatty acids in teleost fish. Reviews in Fisheries Science, 11, 107–184. 10.1080/713610925

[ece39927-bib-0070] Torres‐Ruiz, M. , Wehr, J. D. , & Perrone, A. A. (2010). Are net‐spinning caddisflies what they eat? An investigation using controlled diets and fatty acids. Journal of the North American Benthological Society, 29, 803–813. 10.1899/09-162.1

[ece39927-bib-0071] Twining, C. W. , Bernhardt, J. R. , Derry, A. M. , Hudson, C. M. , Ishikawa, A. , Kabeya, N. , Kainz, M. J. , Kitano, J. , Kowarik, C. , Ladd, S. N. , Leal, M. C. , Scharnweber, K. , Shipley, J. R. , & Matthews, B. (2021). The evolutionary ecology of fatty‐acid variation: Implications for consumer adaptation and diversification. Ecology Letters, 24, 1709–1731. 10.1111/ele.13771 34114320

[ece39927-bib-0072] Twining, C. W. , Brenna, J. T. , Lawrence, P. , Shipley, J. R. , Tollefson, T. N. , & Winkler, D. W. (2016). Omega‐3 long‐chain polyunsaturated fatty acids support aerial insectivore performance more than food quantity. Proceedings of the National Academy of Sciences, USA, 113, 10920–10925. 10.1073/pnas.1603998113 PMC5047183 27638210

[ece39927-bib-0073] Twining, C. W. , Brenna, J. T. , Lawrence, P. , Winkler, D. W. , Flecker, A. S. , & Hairston, N. G., Jr. (2019). Aquatic and terrestrial resources are not nutritionally reciprocal for consumers. Functional Ecology, 33, 2042–2052. 10.1111/1365-2435.13401

[ece39927-bib-0074] Twining, C. W. , Parmar, T. P. , Mathieu‐Resuge, M. , Kainz, M. J. , Shipley, J. R. , & Martin‐Creuzburg, D. (2021). Use of fatty acids from aquatic prey varies with foraging strategy. Frontiers in Ecology and Evolution, 9.

[ece39927-bib-0075] Uetz, G. W. , Bischoff, J. , & Raver, J. (1992). Survivorship of wolf spiders (Lycosidae) reared on different diets. Journal of Arachnology, 20, 207–211.

[ece39927-bib-0076] Vannote, R. L. , Minshall, G. W. , Cummins, K. W. , Sedell, J. R. , & Cushing, C. E. (1980). The river continuum concept. Canadian Journal of Fisheries and Aquatic Sciences, 37, 130–137. 10.1139/f80-017

[ece39927-bib-0077] Wallace, J. B. , Eggert, S. L. , Meyer, J. L. , & Webster, J. R. (1997). Multiple trophic levels of a Forest stream linked to terrestrial litter inputs. Science, 277, 102–104. 10.1126/science.277.5322.102

[ece39927-bib-0078] Wenig, P. , & Odermatt, J. (2010). OpenChrom: A cross‐platform open source software for the mass spectrometric analysis of chromatographic data. BMC Bioinformatics, 11, 405. 10.1186/1471-2105-11-405 20673335 PMC2920884

[ece39927-bib-0079] Wesner, J. S. (2010). Seasonal variation in the trophic structure of a spatial prey subsidy linking aquatic and terrestrial food webs: Adult aquatic insects. Oikos, 119, 170–178. 10.1111/j.1600-0706.2009.17687.x

[ece39927-bib-0080] Wilson, K. , & Cotter, S. C. (2008). Density‐dependent prophy‐ laxis in insects. In D. W. Whitman & T. N. Ananthakrishnan (Eds.), Phenotypic plasticity of insects: Mechanisms and consequences (pp. 137–176). Science Publishers.

[ece39927-bib-0081] Wojda, I. (2017). Temperature stress and insect immunity. Journal of Thermal Biology, 68, 96–103. 10.1016/j.jtherbio.2016.12.002 28689727

[ece39927-bib-0082] Zeis, B. , Buchen, I. , Wacker, A. , & Martin‐Creuzburg, D. (2019). Temperature‐induced changes in body lipid composition affect vulnerability to oxidative stress in *Daphnia magna* . Comparative Biochemistry and Physiology. Part B, Biochemistry & Molecular Biology, 232, 101–107. 10.1016/j.cbpb.2019.03.008 30904725

[ece39927-bib-0083] Zubrod, J. P. , Englert, D. , Wolfram, J. , Rosenfeldt, R. R. , Feckler, A. , Bundschuh, R. , Seitz, F. , Konschak, M. , Baudy, P. , Lüderwald, S. , Fink, P. , Lorke, A. , Schulz, R. , & Bundschuh, M. (2017). Long‐term effects of fungicides on leaf‐associated microorganisms and shredder populations: An artificial stream study. Environmental Toxicology and Chemistry, 36, 2178–2189. 10.1002/etc.3756 28160498

