# Supplementary material for: Trophic transfer of polyunsaturated fatty acids across the aquatic–terrestrial interface: An experimental tritrophic food chain approach
Source: Ecol Evol. 2023 Mar 24;13(3):e9927. doi: 10.1002/ece3.9927 (PMC10037435; doi:10.1002/ece3.9927)
Supplement: Supplementary file 1 — Appendix S1 [file ECE3-13-e9927-s001.pdf]

Supporting information:

Trophic transfer of polyunsaturated fatty acids across the aquatic-terrestrial interface: an experimental tritrophic food chain approach

Katharina Ohler[a], Verena C. Schreiner[a], Dominik Martin-Creuzburg[b], Ralf B. Schäfer [a]

[a] iES Landau, Institute for Environmental Sciences, RPTU Kaiserslautern-Landau, Fortstraße 7, 76829 Landau in der Pfalz, Germany

[b] BTU Cottbus-Senftenberg, Department of Aquatic Ecology, Research Station Bad Saarow, Germany

Table S1: Spiders used in the experiment. T is the abbreviation for the genus *Tetragnatha*. NA: Spiders used at the beginning of the experiment to analyse the polyunsaturated fatty acids (PUFA) profiles, immune response and dry weight.

treatment	experiment	experiment ID	original spider ID	species	sex	adult/ juvenile
NA	1	21	21	*T. montana*	m	a
NA	1	22	22	*T. montana*	m	a
NA	1	24	24	*T. montana*	f	a
NA	1	38	38	*T. montana*	m	a
NA	1	39	39	*T. montana*	f	a
NA	1	44	44	*T. montana*	m	a
NA	1	48	48	*T. montana*	f	a
NA	1	49	49	*T. montana*	f	a
NA	1	59	59	*T. montana*	f	a
NA	1	63	63	*T. montana*	f	a
NA	1	65	65	*T. montana*	f	a
NA	1	70	70	*T. montana*	f	a
NA	1	74	74	*T. montana*	f	a
NA	1	78	78	*T. montana*	f	a
NA	1	79	79	*T. montana*	f	a
NA	1	81	81	*T. montana*	f	a
NA	1	86	86	*T. montana*	f	a
NA	1	106	106	*T. montana*	f	a
NA	1	111	111	*T. montana*	f	a
NA	1	115	115	*T. montana*	f	a
algae	1	algae_1	104	*T. montana*	f	a
algae	1	algae_2	75	*T. montana*	f	a
algae	1	algae_3	40	*T. montana*	f	a
algae	1	algae_4	88	*T. montana*	f	a
algae	1	algae_4	122	*T. montana*	f	a
algae	1	algae_5	82	*T. montana*	f	a
algae	1	algae_6	41	*T. montana*	f	a
algae	1	algae_7	118	*T. montana*	f	a
algae	1	algae_8	89	*T. montana*	f	a
algae	1	algae_9	116	*T. montana*	f	a
algae	1	algae_10	34	*T. montana*	f	a
algae	1	algae_11	114	*T. montana*	f	a
algae	1	algae_12	97	*T. montana*	f	a
algae	1	algae_13	103	*T. montana*	f	a
algae	1	algae_14	62	*T. montana*	f	a
algae	1	algae_15	33	*T. montana*	f	a
algae	1	algae_16	37	*T. montana*	f	a
algae	1	algae_17	100	*T. montana*	f	a
algae	1	algae_18	92	*T. montana*	f	a
algae	1	algae_19	105	*T. montana*	f	a
algae	1	algae_19	124	*T. sp.*	f	j
algae	1	algae_20	77	*T. montana*	f	a
fish food	1	fishfood_1	93	*T. montana*	f	a
fish food	1	fishfood_2	95	*T. montana*	f	a
fish food	1	fishfood_3	55	*T. montana*	f	a
fish food	1	fishfood_4	53	*T. montana*	f	a
fish food	1	fishfood_5	25	*T. montana*	f	a
fish food	1	fishfood_6	23	*T. montana*	f	a
fish food	1	fishfood_7	84	*T. montana*	f	a

Table S1: Continued.

fish food	1	fishfood_7	127	*T. sp.*	f	j
fish food	1	fishfood_8	71	*T. montana*	f	a
fish food	1	fishfood_9	50	*T. montana*	f	a
fish food	1	fishfood_10	107	*T. montana*	f	a
fish food	1	fishfood_11	120	*T. montana*	f	a
fish food	1	fishfood_12	64	*T. montana*	f	a
fish food	1	fishfood_13	121	*T. montana*	f	a
fish food	1	fishfood_14	117	*T. montana*	f	a
fish food	1	fishfood_15	45	*T. montana*	f	a
fish food	1	fishfood_16	108	*T. montana*	f	a
fish food	1	fishfood_17	69	*T. montana*	f	a
fish food	1	fishfood_18	43	*T. montana*	f	a
fish food	1	fishfood_19	52	*T. montana*	f	a
fish food	1	fishfood_20	67	*T. montana*	f	a
oatmeal	1	oatmeal_1	27	*T. sp.*	f	j
oatmeal	1	oatmeal_1	126	*T. montana*	f	a
oatmeal	1	oatmeal_2	85	*T. montana*	f	a
oatmeal	1	oatmeal_3	42	*T. montana*	f	a
oatmeal	1	oatmeal_4	83	*T. montana*	f	a
oatmeal	1	oatmeal_5	54	*T. montana*	f	a
oatmeal	1	oatmeal_5	123	*T. montana*	f	a
oatmeal	1	oatmeal_6	94	*T. montana*	f	a
oatmeal	1	oatmeal_7	80	*T. montana*	f	a
oatmeal	1	oatmeal_8	72	*T. montana*	f	a
oatmeal	1	oatmeal_9	36	*T. montana*	f	a
oatmeal	1	oatmeal_10	60	*T. montana*	f	a
oatmeal	1	oatmeal_11	57	*T. montana*	f	a
oatmeal	1	oatmeal_12	73	*T. montana*	f	a
oatmeal	1	oatmeal_13	113	*T. montana*	f	a
oatmeal	1	oatmeal_14	66	*T. montana*	f	a
oatmeal	1	oatmeal_15	99	*T. montana*	f	a
oatmeal	1	oatmeal_16	68	*T. montana*	f	a
oatmeal	1	oatmeal_17	46	*T. montana*	f	a
oatmeal	1	oatmeal_18	96	*T. montana*	f	a
oatmeal	1	oatmeal_19	56	*T. montana*	f	a
oatmeal	1	oatmeal_20	90	*T. montana*	f	a

Table S1: Continued.

treatment	experiment	experiment ID	original ID	species	sex	adult/ juvenile
NA	2	133	133	*T. montana*	f	a
NA	2	138	138	*T. montana*	f	a
NA	2	163	163	*T. montana*	f	a
NA	2	164	164	*T. montana*	f	a
NA	2	165	165	*T. montana*	f	a
NA	2	168	168	*T. montana*	f	a
NA	2	181	181	*T. montana*	f	a
NA	2	187	187	*T. montana*	f	a
NA	2	202	202	*T. montana*	f	a
NA	2	203	203	*T. montana*	f	a
NA	2	215	215	*T. montana*	f	a
NA	2	216	216	*T. montana*	f	a
NA	2	225	225	*T. montana*	f	a
fish food	2	fishfood_1	198	*T. montana*	f	a
fish food	2	fishfood_1	148	*T. montana*	f	a
fish food	2	fishfood_2	170	*T. montana*	f	a
fish food	2	fishfood_2	147	*T. montana*	f	a
fish food	2	fishfood_3	219	*T. montana*	f	a
fish food	2	fishfood_4	175	*T. montana*	f	a
fish food	2	fishfood_4	136	*T. montana*	f	a
fish food	2	fishfood_4	162	*T. montana*	f	a
fish food	2	fishfood_5	196	*T. montana*	f	a
fish food	2	fishfood_6	180	*T. montana*	f	a
fish food	2	fishfood_7	161	*T. montana*	f	a
fish food	2	fishfood_8	213	*T. montana*	f	a
fish food	2	fishfood_9	158	*T. montana*	f	a
fish food	2	fishfood_10	206	*T. montana*	f	a
fish food	2	fishfood_11	139	*T. montana*	f	a
fish food	2	fishfood_12	214	*T. montana*	f	a
fish food	2	fishfood_12	166	*T. montana*	f	a
fish food	2	fishfood_13	201	*T. montana*	f	a
fish food	2	fishfood_14	131	*T. montana*	f	a
fish food	2	fishfood_15	212	*T. montana*	f	a
fish food	2	fishfood_16	140	*T. montana*	f	a
fish food	2	fishfood_17	185	*T. montana*	f	a
fish food	2	fishfood_18	137	*T. montana*	f	a
fish food	2	fishfood_19	199	*T. montana*	f	a
fish food	2	fishfood_19	177	*T. montana*	f	a
fish food	2	fishfood_20	188	*T. montana*	f	a
leaves	2	leaves_1	207	*T. montana*	f	a
leaves	2	leaves_1	153	*T. montana*	f	a
leaves	2	leaves_2	197	*T. montana*	f	a
leaves	2	leaves_2	184	*T. montana*	f	a
leaves	2	leaves_3	169	*T. montana*	f	a
leaves	2	leaves_4	150	*T. montana*	f	a
leaves	2	leaves_5	152	*T. montana*	f	a
leaves	2	leaves_6	226	*T. montana*	f	a
leaves	2	leaves_7	149	*T. montana*	f	a
leaves	2	leaves_8	142	*T. montana*	f	a
leaves	2	leaves_9	191	*T. montana*	f	a
leaves	2	leaves_10	174	*T. montana*	f	a

Table S1: Continued.

leaves	2	leaves_11	179	*T. montana*	f	a
leaves	2	leaves_11	209	*T. montana*	f	a
leaves	2	leaves_12	193	*T. montana*	f	a
leaves	2	leaves_13	220	*T. montana*	f	a
leaves	2	leaves_14	227	*T. montana*	f	a
leaves	2	leaves_15	183	*T. montana*	f	a
leaves	2	leaves_15	211	*T. montana*	f	a
leaves	2	leaves_16	194	*T. montana*	f	a
leaves	2	leaves_17	144	*T. montana*	f	a
leaves	2	leaves_17	178	*T. montana*	f	a
leaves	2	leaves_18	176	*T. montana*	f	a
leaves	2	leaves_19	210	*T. montana*	f	a
leaves	2	leaves_19	172	*T. montana*	f	a
leaves	2	leaves_20	192	*T. montana*	f	a
leaves	2	leaves_20	208	*T. montana*	f	a
oatmeal	2	oatmeal_1	221	*T. montana*	f	a
oatmeal	2	oatmeal_2	171	*T. montana*	f	a
oatmeal	2	oatmeal_2	182	*T. montana*	f	a
oatmeal	2	oatmeal_2	218	*T. montana*	f	a
oatmeal	2	oatmeal_3	186	*T. montana*	f	a
oatmeal	2	oatmeal_4	159	*T. montana*	f	a
oatmeal	2	oatmeal_5	204	*T. montana*	f	a
oatmeal	2	oatmeal_6	167	*T. montana*	f	a
oatmeal	2	oatmeal_7	222	*T. montana*	f	a
oatmeal	2	oatmeal_8	132	*T. montana*	f	a
oatmeal	2	oatmeal_9	146	*T. montana*	f	a
oatmeal	2	oatmeal_10	190	*T. montana*	f	a
oatmeal	2	oatmeal_11	130	*T. montana*	f	a
oatmeal	2	oatmeal_12	154	*T. montana*	f	a
oatmeal	2	oatmeal_13	189	*T. montana*	f	a
oatmeal	2	oatmeal_14	195	*T. montana*	f	a
oatmeal	2	oatmeal_15	134	*T. montana*	f	a
oatmeal	2	oatmeal_16	223	*T. montana*	f	a
oatmeal	2	oatmeal_17	157	*T. montana*	f	a
oatmeal	2	oatmeal_18	228	*T. montana*	f	a
oatmeal	2	oatmeal_19	135	*T. montana*	f	a
oatmeal	2	oatmeal_19	217	*T. montana*	f	a
oatmeal	2	oatmeal_20	160	*T. montana*	f	a

Table S2: Number of samples used in the PUFA analysis, for dry weight and immune response.

experiment	treatment	time point (days)	trophic level	number samples
1	algae	0	spider	7
1	algae	23	chironomid	4
1	algae	23	basic food source	6
1	algae	23	spider	5
1	algae	44	chironomid	6
1	algae	44	basic food source	6
1	algae	44	spider	3
1	fish food	0	spider	6
1	fish food	23	chironomid	6
1	fish food	23	basic food source	6
1	fish food	23	spider	4
1	fish food	44	chironomid	5
1	fish food	44	basic food source	6
1	fish food	44	spider	3
1	oatmeal	0	spider	7
1	oatmeal	23	chironomid	6
1	oatmeal	23	basic food source	6
1	oatmeal	23	spider	5
1	oatmeal	44	chironomid	6
1	oatmeal	44	basic food source	6
1	oatmeal	44	spider	6
2	fish food	0	spider	4
2	fish food	14	chironomid	4
2	fish food	14	basic food source	4
2	fish food	14	spider	10
2	fish food	21	chironomid	2
2	fish food	21	basic food source	2
2	fish food	21	spider	6
2	leaves	0	spider	5
2	leaves	14	chironomid	5
2	leaves	14	basic food source	4
2	leaves	14	spider	10
2	leaves	21	chironomid	4
2	leaves	21	basic food source	6
2	leaves	21	spider	6
2	oatmeal	0	spider	4
2	oatmeal	14	chironomid	4
2	oatmeal	14	basic food source	5
2	oatmeal	14	spider	10
2	oatmeal	21	chironomid	3
2	oatmeal	21	basic food source	2
2	oatmeal	21	spider	6

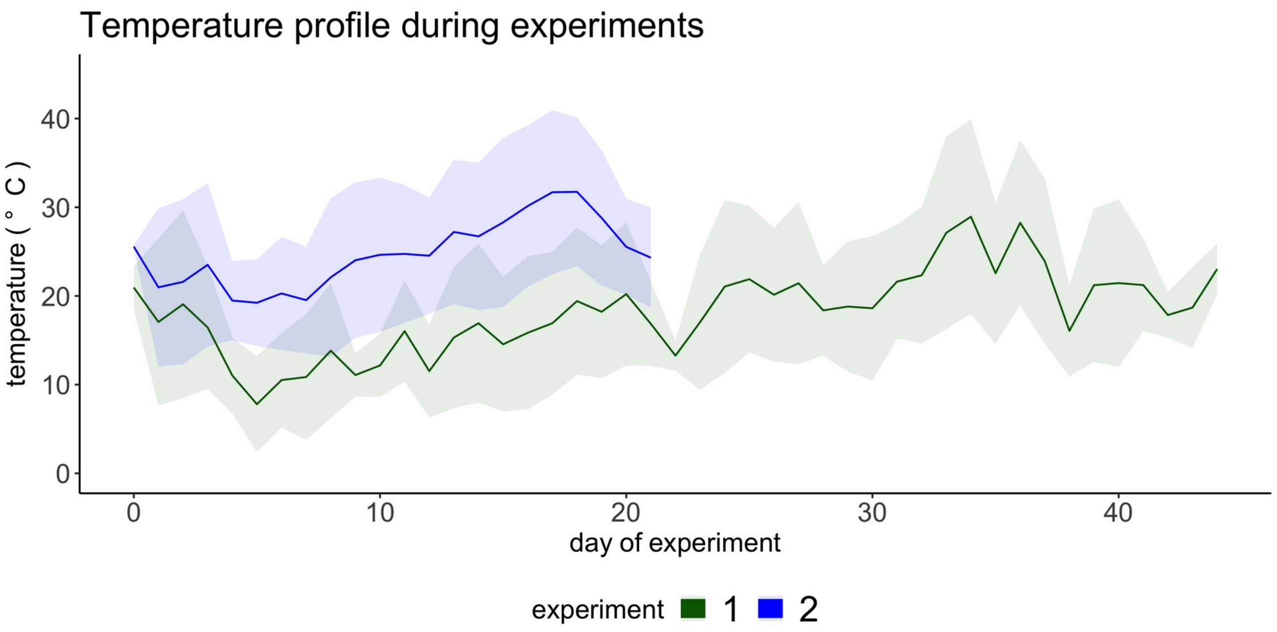

Figure S1: Temperature profiles during the two microcosm experiments. Green: First experiment. Blue: Second experiment. The lines represent the mean temperature per day and the ribbon the standard deviation of the mean temperature per day. The first experiment took place from 29th of April to the 12th of June 2019 and the second experiment was run from the 8th to 29th of July 2019. The first experiment included algae, fish food and oatmeal as basic food treatment and the second experiment included leaves, fish food and oatmeal.

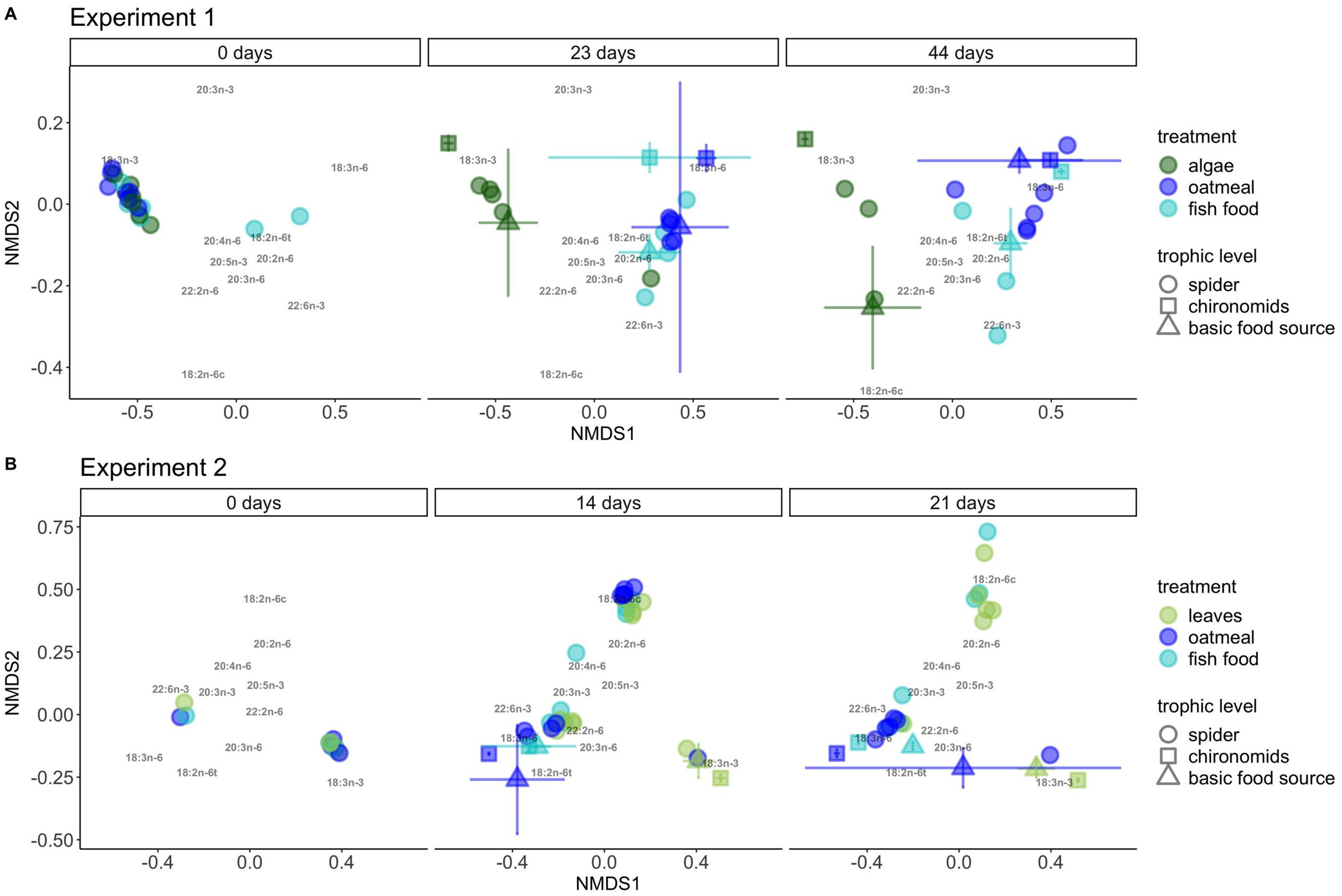

Figure S2: Non-metric multidimensional scaling (NMDS) of the polyunsaturated fatty acid (PUFA) profiles in the first (A) and second (B) experiment. Colours indicate treatment, shape indicates trophic level. The first experiment included algae, fish food and oatmeal as food treatment, whereas the second experiment included leaves, fish food and oatmeal. For the chironomids and basic food sources mean and standard deviation are presented. On day 0, only PUFA profiles of spiders were analysed to gain their starting values before they were fed with chironomids of the different treatments and the spiders were assigned randomly to the treatments. Chironomids and basic food sources were sampled and analysed as soon as the spiders were fed with them. 20:5n-3: eicosapentaenoic acid (EPA), 20:4n-6: arachidonic acid (ARA), 18:3n-3: α-linolenic acid (ALA), 18:2n-6: linoleic acid (LIN), 18:3n-6: γ-linolenic acid (GLA), 18:2n-6t: linolelaidic acid (LLA).

Analytical procedure polyunsaturated fatty acids (PUFA)

In this study, we conducted the extraction of PUFAs following a modified procedure by Folch et al. (1957). PUFAs were extracted in 5 mL chloroform/methanol (v:v; 2:1). After the addition of an internal standard (C17:00 200 µg/mL; C23:0 250 µg/mL, Sigma-Aldrich) the samples were vortexed for 10 s and extracted over night at -20 °C. The amount of internal standard added to the sample depended on the sample weight (Tab. S3).

After the extraction, the samples were filtered with a syringe filter (PTFE, 13 mm, 0.45 µm, BGB), to remove all solid particles. Before the samples were filtered, the syringe filters were washed with chloroform/methanol (v:v; 2:1) three times to reduce baseline noise. The filtered samples were evaporated under nitrogen at 40 °C to dryness and dissolved in methanol during vortexing every sample for 30 s. After 60 s the dissolved samples were transferred to GC-vials. Their headspace was filled with nitrogen and the samples were stored at -20 °C until derivatization to fatty acid methyl esters (FAME).

Methanolic trimethylsulfonium hydroxide (TMSH, 0.2 M, Macherey-Nagel) was used as the derivatization agent, because it was shown to be suitable for PUFA derivatization (Butte, 1983; Gómez-Brandón et al., 2008, 2010). Furthermore, derivatization with TMSH is time-saving, because it is a fast and single-step reaction, which can be done at room temperature (Yamauchi et al., 1979). We pipetted 20 µL of the sample and 10 µL of TMSH in a GC-vial, vortexed for 30 s and then the sample was kept at room temperate until it was measured with a GC-FID (Varian CP-3800, Varian Inc). Nitrogen was the carrier gas with a constant flow of 0.7 mL min^{-1} and 1 µL of the sample was injected splitless at 250 °C. The temperature programme of the column oven is shown in Table S4.

Table S3: Volume of added internal standard and volume of methanol used to dissolve PUFA, depending on the dry weight of the spiders.

**Weight sample (mg)**	**Volume Internal Standard (µL)**	**Volume methanol (µL)**
<13	50	50
30	50	100
45	100	150
60	100	200
75	150	250
90	150	300

Table S4: Temperature programme of the column oven.

**Heating rate (°C min^{-1})**	**Temperature (°C)**	**hold time (min)**
0	60	1
30	150	0
10	180	0
2	205	20
10	220	20

Calculation PUFA content

The samples were measured in three campaigns: January – March 2020, May – June 2020 and November 2020. For every campaign, one calibration curve, limit of quantification (LOQ) and recovery were calculated. For all calculations, R version 4.2.0 (R Core Team, 2022) was used.

The external standards SUPELCO 37 Component FAME mix, ALA- and 18:1n-7-FAME (Sigma-Aldrich) were measured in order to obtain the calibration curve. Failed measurements, measurements with bad quality and outliers were omitted from the calibration. We used linear calibration curves with an intercept of 0 because this increased R^2 of the calibration curves and therefore the quality of the calibration.

The LOQ was determined using blank samples. The blank samples went through the same extraction procedure as the samples but did not contain any PUFA. The mean area of the peaks in the blank samples was compared with the mean area of the peaks in the calibration curves. As LOQ the concentration of standard was set, where the mean peak area in the standard was a factor of three higher than the mean peak area in the blank samples. Concentrations in the samples below the LOQ were set to 0.

The recovery of PUFAs was calculated by dividing the concentration of the internal standard 23:0 measured in the samples by the added concentration of 23:0. The recovery was included in the calculation of the PUFA concentration of the samples. The data and R-Scripts are openly available in GitHub at https://doi.org/10.5281/zenodo.7692685.

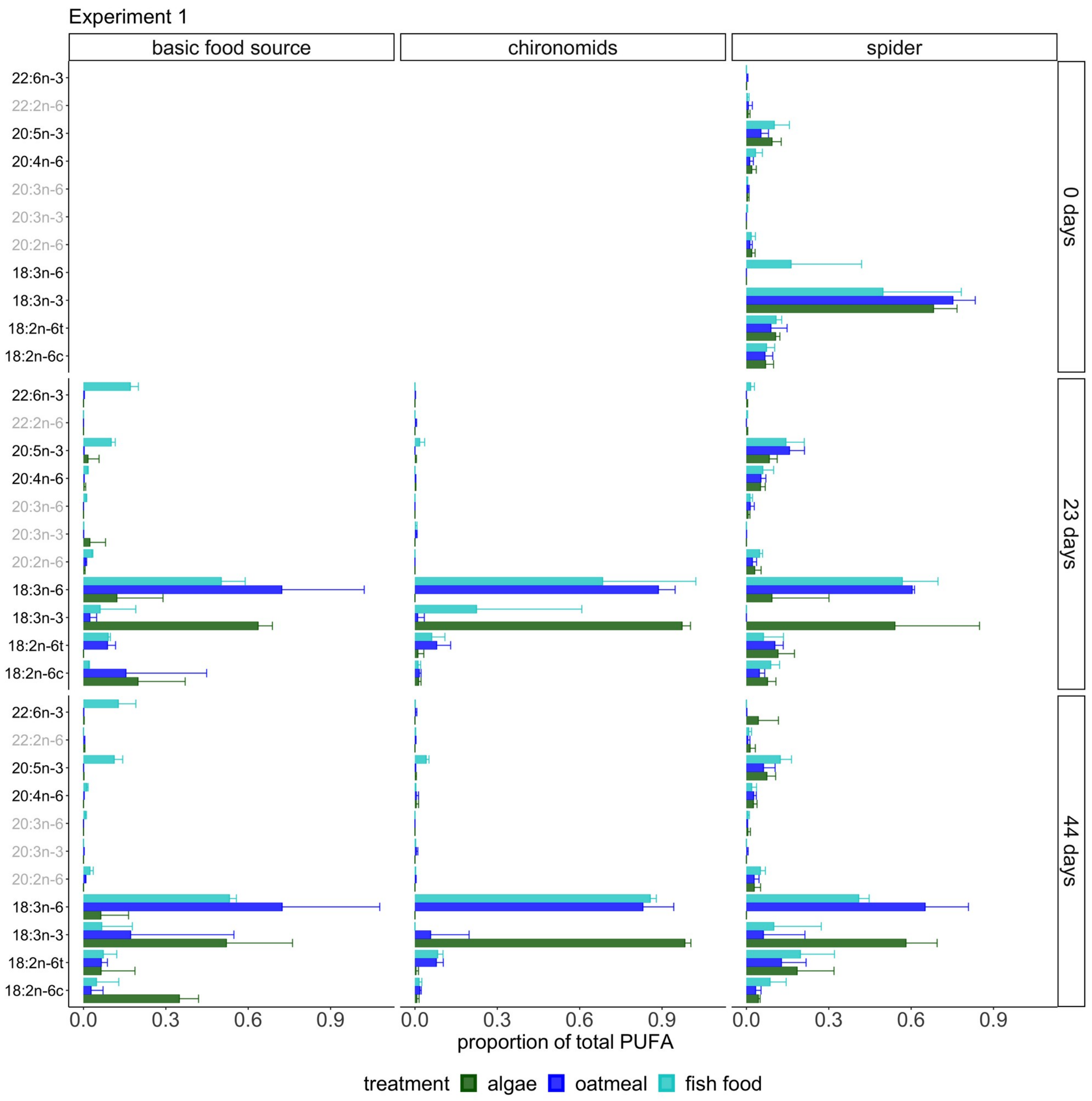

Figure S3: Mean proportion and standard deviation of polyunsaturated fatty acids (PUFA) in the first microcosm experiment. Colours indicate the treatments algae, oatmeal and fish food. On day 0, only PUFA profiles of spiders were analysed to gain their starting values before they were fed with chironomids of the different treatments and the spiders were assigned randomly to the treatments. Eicosapentaenoic acid (EPA, 20:5n-3), arachidonic acid (ARA, 20:4n-6), α-linolenic acid (ALA, 18:3n-3), ɣ-linolenic acid (GLA, 18:3n-6), linolelaidic acid (LLA, 18:2n-6t) and linoleic acid (LIN, 18:2n-6) are written in black and the other PUFA in grey.

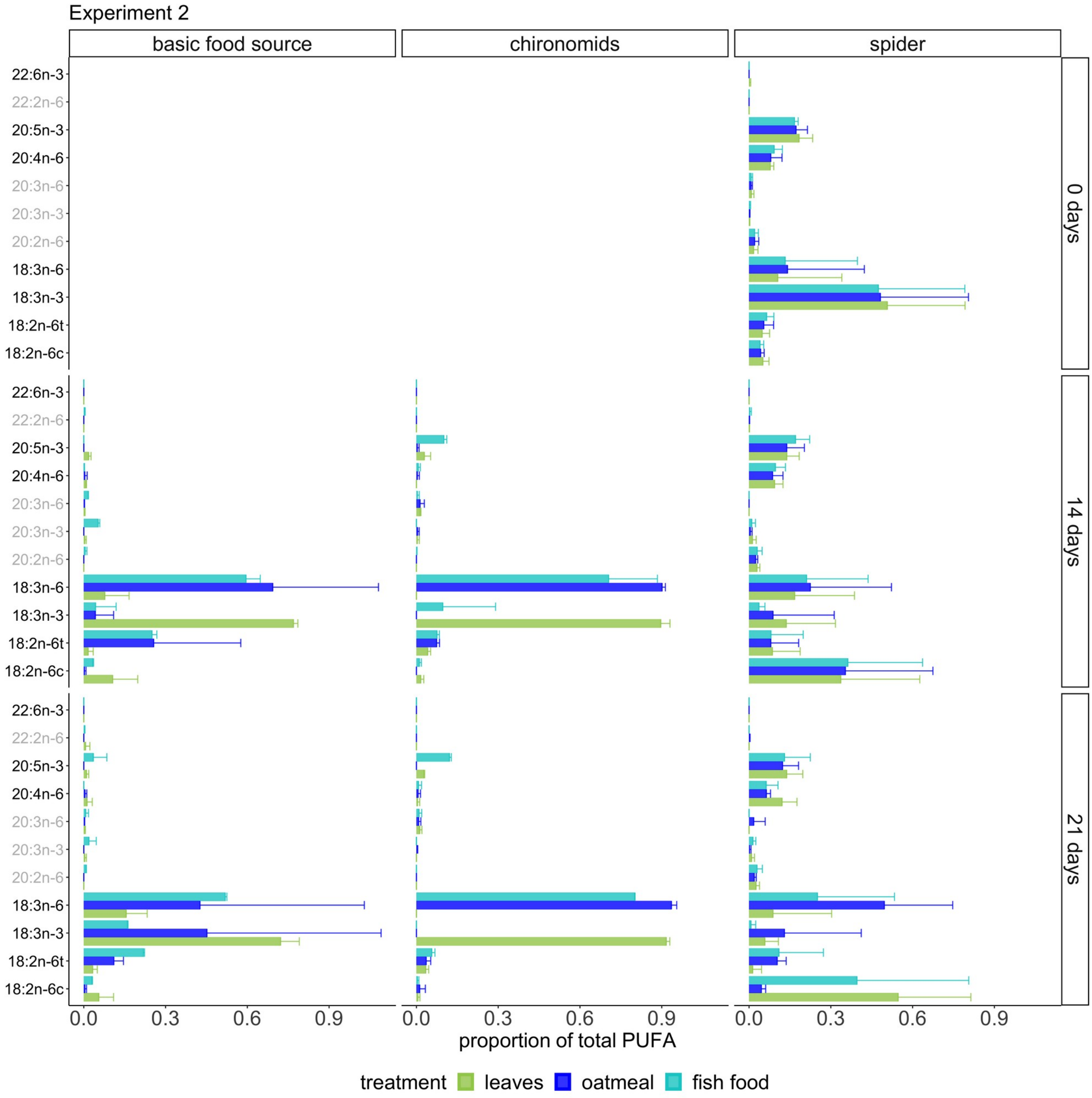

Figure S4: Mean proportion and standard deviation of polyunsaturated fatty acids (PUFA) in the second microcosm experiment. Colours indicate the treatments leaves, oatmeal and fish food. On day 0, only PUFA profiles of spiders were analysed to gain their starting values before they were fed with chironomids of the different treatments and the spiders were assigned randomly to the treatments. Eicosapentaenoic acid (EPA, 20:5n-3), arachidonic acid (ARA, 20:4n-6), α-linolenic acid (ALA, 18:3n-3), ɣ-linolenic acid (GLA, 18:3n-6), linolelaidic acid (LLA, 18:2n-6t) and linoleic acid (LIN, 18:2n-6) are written in black and the other PUFA in grey.

References

Butte, W. (1983). Rapid method for the determination of fatty acid profiles from fats and oils using trimethylsulphonium hydroxide for transesterification. *Journal of Chromatography A*, *261*, 142–145. https://doi.org/10.1016/S0021-9673(01)87931-0

Folch, J., Lees, M., & Stanley, G. H. S. (1957). A SIMPLE METHOD FOR THE ISOLATION AND PURIFICATION OF TOTAL LIPIDES FROM ANIMAL TISSUES. *Journal of Biological Chemistry*, *226*(1), 497–509. https://doi.org/10.1016/S0021-9258(18)64849-5

Gómez-Brandón, M., Lores, M., & Domínguez, J. (2008). Comparison of extraction and derivatization methods for fatty acid analysis in solid environmental matrixes. *Analytical and Bioanalytical Chemistry*, *392*(3), 505–514. https://doi.org/10.1007/s00216-008-2274-7

Gómez-Brandón, M., Lores, M., & Domínguez, J. (2010). A new combination of extraction and derivatization methods that reduces the complexity and preparation time in determining phospholipid fatty acids in solid environmental samples. *Bioresource Technology*, *101*(4), 1348–1354. https://doi.org/10.1016/j.biortech.2009.09.047

R Core Team. (2022). *R: A Language and Environment for Statistical Computing* (4.2.0). R Foundation for Statistical Computing. https://www.r-project.org/

Yamauchi, K., Tanabe, T., & Kinoshita, M. (1979). Trimethylsulfonium hydroxide: A new methylating agent. *The Journal of Organic Chemistry*, *44*(4), 638–639. https://doi.org/10.1021/jo01318a037
